# PRACTICE: Development of a Core Outcome Set for Trials of Physical Rehabilitation in Critical Illness

**DOI:** 10.1513/AnnalsATS.202406-581OC

**Published:** 2024-12-01

**Authors:** Bronwen A. Connolly, Matthew Barclay, Chantal Davies, Nicholas Hart, Natalie Pattison, Gordon Sturmey, Paula R. Williamson, Dale M. Needham, Linda Denehy, Bronagh Blackwood

**Affiliations:** ^1^ Wellcome-Wolfson Institute for Experimental Medicine, Queen’s University Belfast, Belfast, United Kingdom;; ^2^ Lane Fox Clinical Respiratory Physiology Research Centre, Guy’s and St Thomas’ NHS Foundation Trust, London, United Kingdom;; ^3^ Centre for Human and Applied Physiological Sciences, King’s College London, London, United Kingdom;; ^4^ Department of Physiotherapy, The University of Melbourne, Melbourne, Victoria, Australia;; ^5^ Independent ICU Patient Representative, Chislehurst, United Kingdom;; ^6^ School of Health and Social Work, University of Hertfordshire, Hertfordshire, United Kingdom;; ^7^ Department of Critical Care, East & North Hertfordshire NHS Trust, Hertfordshire, United Kingdom;; ^8^ Independent ICU Patient Representative, Thatcham, United Kingdom;; ^9^ MRC-NIHR Trials Methodology Research Partnership, University of Liverpool, Liverpool, United Kingdom; and; ^10^ Division of Pulmonary and Critical Care Medicine and Department of Physical Medicine and Rehabilitation, Johns Hopkins University School of Medicine, Baltimore, Maryland

**Keywords:** physical rehabilitation, consensus, critical illness, outcome, core outcome set

## Abstract

**Rationale:**

Findings from individual trials of physical rehabilitation interventions in critically ill adults have limited potential for meta-analysis and informing clinical decision-making because of the heterogeneity in selection and reporting of outcomes used for evaluation.

**Objectives:**

The objective of this study was to determine a core outcome set (COS) for use in all future trials evaluating physical rehabilitation interventions delivered across the critical illness continuum of recovery.

**Methods:**

An international, two-round, online, modified Delphi consensus process, following recommended standards, was conducted. Participants (*N* = 329) comprised three stakeholder groups—researchers, *n* = 58 (18%); clinicians, *n* = 247 (75%); and patients and caregivers, *n* = 24 (7%)—and represented 26 countries and nine healthcare professions. Participants rated the importance of a range of relevant outcomes. Outcomes included in the COS were those prioritized of “critical importance” by all three stakeholder groups.

**Results:**

Survey response rates were 88% (Round 1) and 91% (Round 2). From a total of 32 initial outcomes, the following outcomes reached consensus for inclusion in the COS: physical function, activities of daily living, survival, health-related quality of life, exercise capacity, cognitive function, emotional and mental well-being, and frailty.

**Conclusions:**

This study developed a consensus-generated COS for future clinical research evaluating physical rehabilitation interventions in critically ill adults across the continuum of recovery. Ascertaining recommended measurement instruments for these core outcomes is now required to facilitate implementation of the COS.

Physical rehabilitation is an essential component in the management of critically ill patients to address impairments in physical function, exercise capacity, and health-related quality of life that are related to post–intensive care syndrome (1). Interventions are recommended across the recovery continuum of critical illness (2), and have been evaluated within the intensive care unit (ICU), after transfer to the ward, after hospital discharge, and across multiple stages of the recovery continuum (3–6). However, heterogeneity in selection and reporting of outcomes in trials evaluating physical rehabilitation interventions in critical illness limits the interpretation of individual study findings and precludes synthesis of multiple datasets (7). For example, four of the most recently published international trials of physical rehabilitation in the ICU each reported a different primary outcome and had total number of outcomes (primary and secondary) ranging between 7 and 18 (3, 8–10). Only the outcome of physical function was consistent across the four trials, albeit with variability in outcome measure and timing of data collection. Generic function was measured using the World Health Organization Disability Assessment Schedule at Day 180 (3) and functional independence was measured using ambulation and the Functional Independence Measure at hospital discharge and at 1 year (8), the peak modified ICU Mobility Scale within 48 hours of ICU discharge (9), and the Physical Function Test for ICU-scored at 3 days after ICU discharge (with other measures of physical function also reported) (10). This heterogeneity fosters outcome-reporting bias and research waste (11, 12) and reduces the usefulness of trials for informing evidence-based clinical decision-making in this area (13). Lack of consensus exists on the most appropriate outcomes for use in these trials (14).

Core outcome sets (COSs) represent an approach for helping address the aforementioned issues. A COS is an agreed-on collection of outcomes to be measured and reported, as a minimum, in all clinical trials for a defined field of interest (11, 12). The value of a COS lies in harnessing consistency in all trials measuring a minimum set of identical outcomes; applying a COS to future trials would generate this consistency for facilitating data synthesis. Examples of existing COSs in critical illness include long-term outcomes after hospital discharge in survivors of acute respiratory failure (15), mechanical ventilation (16), cardiac arrest (17), and delirium (18). However, no COS exists for physical rehabilitation in critically ill adults.

Therefore, the aim of the present study PRACTICE (Physical Rehabilitation Core Outcomes in Critical Illness) was to develop a COS for trials of physical rehabilitation interventions delivered across the continuum of recovery for critically ill adults. Specifically, the scope of the COS primarily relates to quantitative clinical research studies that evaluate physical rehabilitation interventions (e.g., mobilization, exercise, or adjuncts such as cycling or electrical muscle stimulation) delivered to adult critically ill patients at one or more stages of the recovery continuum (i.e., in the ICU, on the hospital ward, or after hospital discharge).

PRACTICE was registered *a priori* on the COMET database (Record ID 288; http://www.comet-initiative.org/Studies/Details/288).

An initial version of the results was presented in abstract form at the 2019 American Thoracic Society International Conference (https://www.atsjournals.org/doi/abs/10.1164/ajrccm-conference.2019.199.1_MeetingAbstracts.A4112).

## Methods

We conducted an international, two-round, online, modified Delphi consensus process to determine the core outcomes (“what” to measure) for the PRACTICE COS. Our methods align with recommended COS development and reporting (19–21); the study protocol has been published (22), with the details reported elsewhere (*see* the data supplement). In brief, we recruited a large, diverse, international participant panel representing three stakeholder groups of “researchers,” “clinicians,” and “patients and caregivers” (for full details of the recruitment processes, *see* Section E1 in the data supplement). Participants rated the importance of a range of outcomes (*see* Section E2 and Table E1) that were sourced through prior systematic reviews of quantitative (23) and qualitative (24) literature, revised and refined by the study team, and supported by findings from patient and care partner interviews (25). Participants were reminded that the goal of the PRACTICE COS was to determine the minimum set of outcomes for evaluation in all future trials of physical rehabilitation in critically ill adults across the recovery continuum. The two-round Delphi process commenced on June 21, 2018 and was completed on September 14, 2018.

Each outcome was rated using the Grading of Recommendations Assessment, Development and Evaluation (or, GRADE) scale (26), with scores ranging from 1 to 9 in terms of importance for inclusion in the final COS (1–3, “not important” for inclusion; 4–6, “important” but not critical; and 7–9, “critical” for inclusion). Participants were also provided with an “unable to score” response if they considered themselves unable to rate an outcome. Consensus for determining the importance of an outcome by a particular stakeholder group was defined as ≥70% of responses rating the outcome as “critical,” and ≤15% of responses rating the outcome as “not important.” Core outcomes were those agreed on by all three stakeholder groups using these consensus criteria (27). Only participants who fully completed Round 1 were invited to complete Round 2, where additional outcomes suggested by participants (from Round 1) were added to the consensus survey, and the wording of several outcomes was revised for clarity (*see* Section E3 and Tables E2 and E3). In Round 2, participants were provided with feedback on Round 1 scoring in the form of histograms for the whole participant panel and for each stakeholder group and were shown their previous individual score for each Round 1 outcome. Rescoring was requested on the basis of this feedback, with rationale requested in the event that any change of score altered the overall category of importance rating (*see* Section E4 of the data supplement). In both rounds, the order of outcomes was randomized to one of four different orders.

DelphiManager software (COMET Initiative) was used to administer the consensus survey rounds. Response rates were defined as the proportion of recruited participants who completed each survey round out of the total number of participants for that stakeholder group. Rates were reported for each stakeholder group. Descriptive statistics were used to analyze and summarize survey round responses, using GraphPad Prism Version 7.0days (GraphPad Software; www.graphpad.com). Histograms and other data management were conducted using Microsoft Excel (Microsoft Office). PRACTICE was registered *a priori* on the COMET database (Record ID 288; http://www.comet-initiative.org/Studies/Details/288) and follows recommendations for COS development (20) and reporting (21). Confidentiality was ensured by allocation of a unique identifier to each participant and data storage on secure, encrypted, password-protected, institutional devices. Participant information sheets were circulated during the promotion of the project, and the landing page of the survey also included information regarding participation. Completion and submission of the electronic surveys indicated consent to participate. The study was approved by the King’s College London BDM (Biomedical Sciences, Medicine, Dentistry and Natural and Mathematical Sciences) Research Ethics Panel (LRS-17/18-4603), and the UK Health Research Authority National Research Ethics Service North-East Committee (18/NE/0018).

## Results

A total of 329 participants completed Round 1, representing 88% of 376 eligible expressions of interest who received the survey link. The panel comprised 58 researchers (18%), 247 clinicians (75%), and 24 patients and caregivers (7%). Participant details are provided in Table 1 (*see* also Section E5 and Table E4). The mean age of participants was 44 years (SD = 10), and the majority of participants were female (*n* = 193; 59%). Participants represented 26 countries—most from the United Kingdom (*n* = 193; 59%)—and nine professions. Within the researcher stakeholder group, the largest professional group comprised physicians (*n* = 30; 52%), whereas the largest professional group within the clinician stakeholder group comprised physical therapists (*n* = 119; 48%). Mean years of professional experience were 22 (SD = 10) and 18 (SD = 8) for the researcher and clinician stakeholder groups, respectively. Almost all researchers and clinicians managed patients in the ICU, with lesser involvement with patients after critical illness on the ward and after hospital discharge. Most patient and caregiver participants (*n* = 17; 71%) were discharged from the ICU less than 3 years prior to participation in the PRACTICE COS study.

**Table 1.** Participant characteristics

Characteristic	Researchers (*n* = 58)	Clinicians (*n* = 247)	Patients and Caregivers (*n* = 24)
Female, *n* (%)*	24 (41)	151 (61)	18 (75)
Age, yr, mean (SD)^†^	47 (10)	42 (8)	54 (13)
Region of residence, *n* (%)^‡^			
United Kingdom	15 (26)	167 (67)	11 (46)
North America	12 (21)	27 (11)	12 (50)
Europe	15 (26)	27 (11)	0
Australasia	10 (17)	13 (5)	1 (4)
South America	4 (7)	4 (2)	0
Africa	0	6 (2)	0
Asia	2 (3)	3 (1)	0
Occupation, *n* (%)^§^			
PT	17 (29)	119 (48)	N/A
Physician	30 (52)	80 (32)	N/A
Nurse	6 (10)	23 (9)	N/A
SLT/P	0	10 (4)	N/A
Dietitian	0	8 (3)	N/A
OT	0	5 (2)	N/A
Other	5 (9)	2 (<1)	N/A
Professional experience, yr, mean (SD)^‖^	22 (10)	18 (8)	N/A
Professional involvement with patients, *n* (%)^¶^			
In the ICU	55 (95)	243 (98)	N/A
Ward based	18 (31)	112 (45)	N/A
Posthospital	21 (36)	49 (20)	N/A
Years since ICU discharge, *n* (%)			
0 to ≤3	N/A	N/A	17 (71)
>3 to ≤6	N/A	N/A	1 (4)
>6 to ≤9	N/A	N/A	2 (8)
9+	N/A	N/A	4 (17)

*Definition of abbreviations*: CTU = clinical trials unit; ICU = intensive care unit; N/A = not applicable; OT = occupational therapist; PT = physical therapist/physiotherapist; SLT/P = speech and language therapist/pathologist.

Note that percentages are rounded to the nearest whole and, therefore, may not total 100.

Date are reported as *n* (%) or mean (SD).

* *n* = 327; 2 participants (*n* = 2 from the clinician stakeholder group) did not report sex.

^†^ *n* = 324; 5 participants (1 from the researcher stakeholder group and 4 from the clinician stakeholder group) did not report age.

^‡^ *n* = 26 individual countries represented (for further details, *see* Table E4).

^§^ *n* = 7; “other” professions included CTU researchers (*n* = 3), nurse practitioner (*n* = 1), and respiratory therapist (*n* = 1).

^‖^ Seven participants (4 from the researcher stakeholder group and 3 from the clinician stakeholder group) did not report duration of professional experience.

^¶^ Respondents could select more than one option related to working across more than one setting.

### Round 1

Thirty outcomes were included in Round 1 (Section E2 and Table E1). Four outcomes reached consensus for inclusion as outcomes in the core set: activities of daily living, physical function, health-related quality of life, and survival (Table 2). A full breakdown of scoring for each outcome according to stakeholder group is reported elsewhere (*see* Section E6 and Table E5). Panel members suggested 51 additional outcomes for consideration in Round 2 (Section E3 and Table E2); after removal of duplicate and nonrelevant outcomes, this resulted in the addition of two new unique outcomes to Round 2 (resilience and bone health). All other outcomes from Round 1 were carried forward into Round 2.

**Table 2.** Consensus results for Round 1 outcome scoring

Outcome	Proportion of Participants Scoring Outcome 7–9*			
	All Participants (*n* = 329)^†^	Researchers (*n* = 58)	Clinicians (*n* = 247)	Patients and Caregivers (*n* = 24)
Consensus met				
Physical function	313 (95)	56 (97)	236 (96)	21 (88)
Activities of daily living	290 (88)	50 (86)	221 (90)	19 (80)
Survival	265 (81)	49 (85)	194 (79)	22 (91)
Health-related quality of life	264 (80)	45 (78)	202 (82)	17 (71)
Consensus not met				
Cognitive function	246 (75)	39 (67)	188 (76)	19 (79)
Return to work or prior role	228 (69)	36 (62)	180 (73)	12 (50)
Exercise capacity	225 (68)	38 (66)	171 (69)	16 (67)
Duration of mechanical ventilation	220 (67)	40 (69)	164 (66)	16 (67)
Frailty	215 (65)	38 (66)	159 (64)	18 (75)
Fatigue	215 (65)	37 (64)	163 (66)	15 (62)
Emotional and mental well-being	214 (65)	37 (64)	160 (65)	17 (71)
Delirium and related symptoms	209 (64)	36 (62)	159 (64)	14 (58)
Healthcare resource utilization	206 (63)	35 (60)	154 (62)	17 (71)
Respiratory (pulmonary) function and symptoms	202 (61)	27 (47)	155 (63)	20 (83)
Place of residence	199 (61)	36 (62)	150 (61)	13 (54)
Muscle and/or motor nerve function	189 (57)	30 (52)	143 (58)	16 (67)
Communication difficulties	185 (56)	23 (40)	141 (57)	21 (88)
Swallowing function and symptoms	182 (55)	30 (52)	134 (54)	18 (75)
Pain	166 (51)	27 (47)	126 (51)	13 (54)
Patient experience of physical rehabilitation	163 (50)	18 (31)	128 (52)	17 (71)
Successful extubation	156 (47)	25 (43)	114 (46)	17 (71)
Reintubation	143 (44)	20 (35)	112 (45)	11 (46)
Social roles, activities, or relationships	137 (42)	21 (36)	105 (43)	11 (46)
Sleep and related symptoms	129 (39)	19 (33)	95 (39)	15 (62)
Nutrition-related parameters	104 (32)	16 (28)	80 (32)	8 (33)
Joint function	91 (28)	16 (28)	63 (26)	12 (50)
Financial impact on patient	87 (26)	18 (31)	57 (23)	12 (50)
Urinary function	57 (17)	7 (12)	38 (15)	12 (50)
Gastrointestinal symptoms	54 (16)	8 (14)	36 (15)	10 (42)
Sexual function	46 (14)	7 (12)	53 (22)	5 (21)

* Each outcome was scored according to the Grading of Recommendations Assessment, Development and Evaluation (or, GRADE) scale (25), ranging from 1 to 9 in terms of importance for inclusion in the final core outcome set (1–3, not important for inclusion; 4–6, important but not critical; and 7–9, critical for inclusion). Consensus for inclusion of an outcome by a particular stakeholder group was defined as ≥70% of responses rating the outcome as “critical,” and ≤15% of responses rating the outcome as “not important.” Consensus for an outcome included in the core outcome set was defined as all three stakeholder groups scoring the outcome as critical for inclusion. Outcomes are ordered by the proportion of all participants scoring 7–9 according to meeting, and not meeting, consensus. The maximum number of participants who indicated “unable to score” for any outcome was 4. For a full scoring breakdown, *see* Table E5.

^†^ Data are reported as *n* (%).

### Round 2

**Figure 1.**
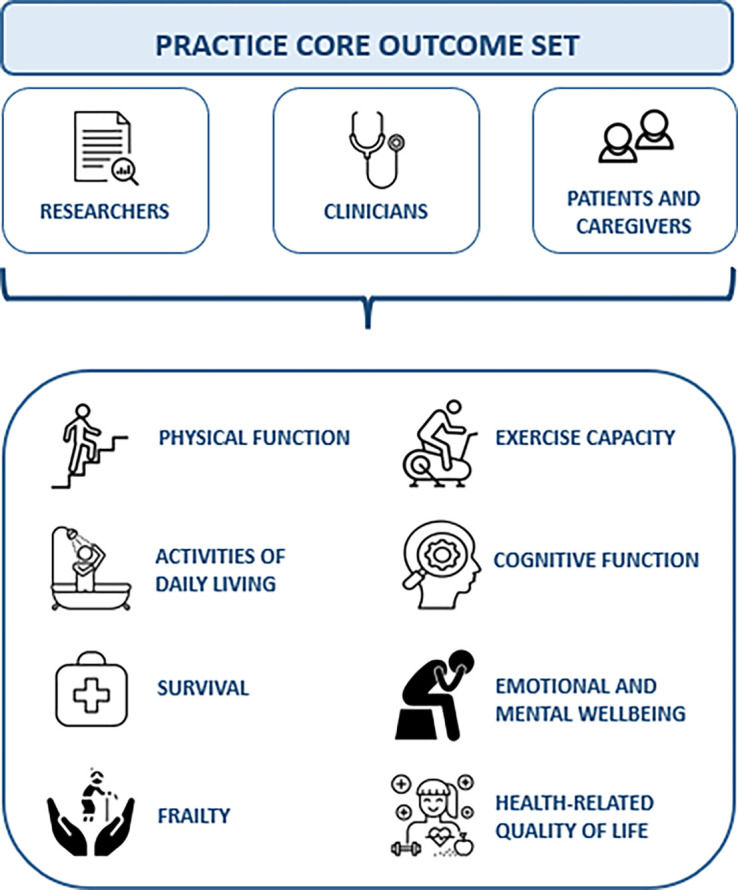
The Physical Rehabilitation Core Outcomes in Critical Illness (PRACTICE) core outcome set. These are the eight outcomes agreed on as critically important for inclusion in the core outcome set by all three stakeholder groups (researchers, clinicians, and patients and caregivers) and forming the core outcome set. This core outcome set can be used for future clinical research trials in physical rehabilitation for critically ill patients across the recovery continuum.

Three hundred participants (91% of the participants in Round 1) completed Round 2. The panel comprised 55 researchers (18% of the participants in Round 2; 95% of the researchers in Round 1), 226 clinicians (75% of the participants in Round 2; 91% of the clinicians in Round 1), and 19 patients and caregivers (6% of the participants in Round 1; 79% of the participants in Round 1). The results are summarized in Table 3 (for more details, *see* Section E7 and Tables E6 and E7). All four outcomes reaching consensus for the COS from Round 1 (activities of daily living, physical function, health-related quality of life, and survival) retained agreement for inclusion with increased support. One outcome (physical function) scored 100% for critical importance by all participants. Neither of the two additional outcomes suggested by participants in Round 1 met the consensus criteria (resilience, 40%; bone health, 16%). Four additional outcomes (exercise capacity, cognitive function, emotional and mental well-being, and frailty) were scored as critically important by all three stakeholder groups. The final COS is presented in Figure 1.

**Table 3.** Consensus results for Round 2 outcome scoring

Outcome	Proportion of Participants Scoring Outcome 7–9*			
	All Participants (*n* = 300)^†^	Researchers (*n* = 55)	Clinicians (*n* = 226)	Patients and Caregivers (*n* = 19)
Consensus met				
Physical function	299 (100)	55 (100)	225 (100)	19 (100)
Activities of daily living	295 (98)	54 (98)	224 (99)	17 (90)
Survival	276 (92)	54 (98)	204 (90)	18 (95)
Health-related quality of life	268 (90)	47 (86)	209 (93)	15 (79)
Exercise capacity	253 (84)	45 (82)	192 (85)	16 (84)
Cognitive function	251 (83)	41 (75)	194 (86)	16 (84)
Emotional and mental well-being	232 (78)	41 (75)	178 (79)	15 (79)
Frailty	227 (76)	41 (75)	168 (74)	18 (95)
Consensus not met				
Duration of mechanical ventilation	230 (77)	42 (76)	176 (78)	12 (63)
Return to work or prior role	229 (76)	38 (69)	181 (80)	10 (53)
Fatigue	229 (76)	46 (84)	172 (76)	11 (58)
Respiratory (pulmonary) function and symptoms	221 (74)	31 (56)	172 (76)	18 (95)
Healthcare resource utilization	214 (71)	39 (71)	162 (72)	13 (68)
Delirium and related symptoms	211 (70)	38 (69)	162 (72)	11 (58)
Place of residence	210 (70)	38 (69)	160 (71)	12 (63)
Muscle and/or motor nerve function	202 (67)	37 (67)	151 (67)	14 (74)
Swallowing function and symptoms	192 (64)	34 (62)	142 (63)	16 (84)
Communication difficulties	184 (61)	27 (49)	140 (62)	17 (90)
Patient experience of physical rehabilitation	163 (54)	22 (40)	127 (56)	14 (74)
Pain	155 (52)	24 (44)	120 (53)	11 (58)
Successful extubation	147 (49)	25 (46)	111 (50)	11 (58)
Reintubation	147 (49)	25 (46)	112 (50)	10 (53)
Social roles, activities or relationships	116 (39)	15 (27)	90 (40)	11 (58)
Sleep and related symptoms	111 (37)	17 (31)	85 (38)	9 (48)
Joint function	63 (21)	12 (22)	42 (19)	9 (47)
Nutrition-related parameters	60 (20)	9 (16)	44 (20)	7 (37)
Financial impact on patient	59 (20)	15 (27)	32 (14)	12 (63)
Urinary function	32 (11)	3 (5.5)	21 (10)	8 (42)
Gastrointestinal symptoms	32 (11)	5 (9)	21 (9)	6 (32)
Sexual function	30 (10)	6 (11)	19 (9)	5 (26)
Additional outcomes from Round 1				
Resilience	121 (40)	19 (35)	88 (39)	14 (74)
Bone health	49 (16)	10 (18)	32 (14)	7 (37)

* Each outcome was scored according to the Grading of Recommendations Assessment, Development and Evaluation (or, GRADE) scale (25), ranging from 1 to 9 in terms of importance for inclusion in the final core outcome set (1–3, not important for inclusion; 4–6, important but not critical; and 7–9, critical for inclusion). Consensus for inclusion of an outcome by a particular stakeholder group was defined as ≥70% of responses rating the outcome as “critical,” and ≤15% of responses rating the outcome “not important.” Consensus for an outcome included in the core outcome set was defined as all three stakeholder groups scoring the outcome as critical for inclusion. Outcomes are ordered by the proportion of all participants scoring 7–9 according to meeting, and not meeting, consensus. The maximum number of participants who indicated “unable to score” for any outcome was 5. For a full scoring breakdown, *see* Table E6.

^†^ Data are reported as *n* (%).

## Discussion

This study has determined a COS for future trials of physical rehabilitation in critically ill adults across the recovery continuum by means of a rigorous international online modified Delphi consensus process. All three stakeholder groups—researchers, clinicians, and patients and caregivers—prioritized eight core outcomes that reflect a broad range of the impairments experienced by adult survivors of critical illness: physical function, activities of daily living, survival, health-related quality of life, exercise capacity, cognitive function, emotional and mental well-being, and frailty.

### Clinical Interpretation

Given the scope of this COS, which was focused on outcomes for physical rehabilitation interventions in critically ill adults, it was relevant that results included multiple outcomes related to physical performance or ability; three were ultimately identified— physical function, activities of daily living, and exercise capacity. Indeed, the inclusion of physical function was unanimous. In addition, although health-related quality of life is an outcome that encapsulates multiple elements, it typically includes some reflection of the impact of an individual’s physical status, and physical impairment is a contributor to poor health-related quality of life in survivors of critical illness up to 10 years thereafter (28). The importance of the interaction between physical and cognitive health after critical illness is recognized; a recent randomized trial reported a significant reduction in cognitive impairment at 1 year after hospital discharge in patients who received an early rehabilitation cotreatment by a physiotherapist and occupational therapist (24%; 24/99 participants) compared with usual care (43%; 43/99 participants), absolute difference, −19%; 95% confidence interval, −32 to −6, *P* = 0.0043) (8).

Early mobilization interventions in the ICU have been systematically reviewed, concluding no association with mortality compared with usual care (relative ratio, 0.98; 95% confidence interval, 0.87 to 1.12; *P* = 0.81) (29). However, inclusion of survival as an outcome in PRACTICE mirrors other COSs in critically ill populations (15, 16, 18), and capturing these data is important for trial process-reporting of patient flow. Reporting attribution of mortality to a physical rehabilitation intervention is essential; a previous trial of functional electrical stimulation in conjunction with in-bed cycle ergometry showed 85% of decedents allocated to the intervention group had never received the intervention before death (30). Physical health and emotional and mental well-being are closely interrelated when outcomes of critical care survivors are examined; poor physical functioning and independence are associated with worse mental health (31), and coexistence of physical impairment and mental health symptoms is common (32). Frailty is a complex syndrome that can be characterized through distinct models—for example, a physical phenotype, an accumulated deficits model across multiple domains, and a multidimensional approach capturing holistic impairment (33). It is interesting that this was the outcome which, although meeting criteria for inclusion in the COS, showed greatest variation in ratings between researcher, clinician, and patient and caregiver stakeholders. Frailty is associated with increased post-ICU disability (34). That this was rated so highly by patients and caregivers (95% rated it as critically important) may reflect the personal perspective of these participants and the cumulative impact of multiple sequelae after their critical illness, and it is important for researchers and clinicians to be cognizant of this.

Beyond the core outcomes that were agreed on, the outcomes that individual stakeholder groups considered important are valuable for the appreciation of their different perspectives with regard to physical rehabilitation interventions. For example, patients and caregivers highly rated the experience of participating in rehabilitation, a finding that should focus researchers and clinicians on how interventions are designed and delivered to maximize engagement, adherence, and fidelity. Furthermore, clinicians highly rated delirium, perhaps influenced by their knowledge and experience of how this condition can impact patients’ ability to participate in rehabilitation activities in the ICU. In addition, the role of clinicians in discharge planning and rehabilitation requirements at later recovery stages may have contributed to their views on the importance of place of residence and return to work or prior role as outcomes.

Outcomes prioritized in the PRACTICE COS reflect three of the areas highlighted by the COMET taxonomy: death, life impact, and physiological/clinical (35), with resource use and adverse events not represented in the final COS. Resource use typically captures economic data, which may be assessed separately in physical rehabilitation trials through parallel health economic analyses—for example, (36, 37)—or outcomes related to hospital admission (e.g., ICU and hospital length of stay, or mechanical ventilation duration) and/or the need for concomitant interventions such as other organ support or medications (35). The latter data are frequently captured as baseline characteristic features of populations enrolled into physical rehabilitation trials. The final domain within resource use is that of societal/carer burden, which captures outcomes relating to the financial or time implications on individual carers or society (35). The impact on families and caregivers after a patient’s critical illness is increasingly appreciated (38–41). The inclusion of outcomes related to caregiver burden as a result of physical rehabilitation interventions delivered to patients may be important to consider in future trials to ensure a holistic approach. With regard to the area of adverse events, these data are typically defined during protocol development, specific to an individual trial, and reported as part of clinical trial conduct (42), thereby removing the need for inclusion as a core outcome.

Finally, the scope of the PRACTICE COS was defined as physical rehabilitation interventions in critically ill adults across the recovery continuum, agnostic to any specific clinical condition or patient population. However, we observed considerable overlap in core outcomes with COS focused on other aspects of managing critically ill patients such as long-term outcomes, nutrition and metabolic interventions, and delirium, which reflects the homogeneity of key features of survivorship; for example, survival (15, 18, 43), physical function (15, 43), cognition, health-related quality of life, and emotional and mental well-being (15, 18), and activities of daily living (43). Nonetheless, this still does not preclude researchers from referring to other bespoke COSs to select outcomes where a particular context is warranted; for example, patients with COVID-19 (44), with cardiac arrest (17), or receiving extracorporeal membrane oxygenation (45).

### Critique of the Method

This study benefited from rigorous methods that followed published recommendations for COS development and reporting (20, 21), were published *a priori* (22), and were consistent with those adopted by similar studies in critical care (15–18). Our participant panel was large, international, and multiprofessional. That said, despite wide attempts to engage participation in as many countries as possible, some regions are relatively underrepresented where contact details were difficult to obtain. In addition, organizational policy precluded the circulation of study information to local memberships of some professional societies, which limited dissemination of the project through these channels. Our survey was limited to the English language, and the predominance of participants from high-income countries may reflect responses from those with differential experience of greater access to rehabilitation services. Our patient and caregiver stakeholder group was modest in size in comparison with the researcher and clinician groups, although this is similarly observed in other critical care COSs (16, 45). Despite recognition of the importance of patient and public involvement in COS development (46), this remains a challenging stakeholder group to recruit, for various reasons (47). However, we ensured that their voice was equally balanced with those of the other two stakeholder groups, thereby avoiding potential bias from results on the basis of the size of stakeholder group and ensuring that our results reflect outcomes that are meaningful to patients and caregivers (48, 49). Our study also benefited from the input of two former ICU patients within the study team (C.D. and G.S.) (19, 47). Notably, we had minimal participant attrition, with more than 90% of researchers and clinicians and nearly 80% of patients and caregivers participating in both survey rounds.

The list of outcomes presented in Round 1 was comprehensive and widely sourced. We initially planned to present outcomes in the consensus survey rounds according to which stage of the recovery continuum after critical illness they had reportedly been evaluated (22). However, we found that this was not necessary, as outcomes typically featured across multiple stages; therefore, we elected instead to present outcomes as one whole list and randomized into four different orders with the benefit of avoiding potential response order bias (50). We had also anticipated for a third consensus survey round to enable two rounds of importance rating for any additional outcomes introduced through Round 1. However, as there were only two of these outcomes, both scoring low for importance rating, a third consensus round was unnecessary. We ensured clear participant information through the consensus survey rounds that reinforced the purpose and scope of the PRACTICE COS. However, some participants’ responses may still have been informed by prior preferences on items they felt were important or related to (physical) recovery overall after critical illness, rather than as an outcome to evaluate effectiveness of a physical rehabilitation intervention *per se*.

Our data collection predates the coronavirus disease (COVID-19) pandemic; therefore considering the stability of our findings during the interim and their contemporary representativeness with current reporting is important. COSs are not typically updated on a frequent basis; updated COSs from many other clinical conditions (there are no known updates to critical care-related COSs) have, on average, occurred approximately 15 years after first development, with the majority nearer to 20 years (51–58). The predominant reason for updating is to reflect advancements in COS methodology (e.g., enhanced inclusion of patient and public partners). Relatively earlier updates have been in response to significant treatment advancements in the field that impact potential clinical and patient-reported outcomes, such as novel immuno- and targeted therapy in lung cancer (52). The robust approach to the development of the PRACTICE COS supports its methodological longevity and rigor. Furthermore, its external validity can be evidenced in the three trials of physical rehabilitation interventions described earlier. These were published subsequent to PRACTICE data collection and continue to demonstrate outcome heterogeneity. However, it is important to note that all individual primary and secondary outcomes were reflected in outcomes included for rating in the PRACTICE Delphi consensus process (3, 8, 9). The mixed findings from these studies, and other physical rehabilitation studies to date, highlight challenges in the interpretation of different rehabilitation *interventions*, but their choice of *outcomes* used for evaluation indicate stability in the representativeness and reliability of the PRACTICE COS.

The PRACTICE COS may not immediately impact the ability to synthesize data across existing trials of physical rehabilitation interventions in critically ill adults, unless they report any of the core outcomes and have commonality in outcome measures and timing of data collection. The true value of the COS lies in encouraging future trials to refer to and adopt it when designing their trial protocols, and the exponential application of the COS in this way would result in greater consistency and synthesis across studies. Determining consensus on measurement variables for the core outcomes is vital if the COS is to be successfully implementable and is the focus of the next stage of the PRACTICE study. As part of this, existing measures, tools, or instruments will be identified as potential candidates, and a further Delphi process conducted. It is important to note that, given the similarity across many existing COSs in critical illness, regardless of intervention, overlapping outcomes where agreement has already been achieved for measurement will be reviewed and considered for PRACTICE (e.g., health-related quality of life and emotional and mental well-being). In this way, unnecessary duplication of efforts will be avoided, and participant effort will be focused on those core outcomes in PRACTICE without agreed-on outcome measures. In the future, the development of COS for other aspects of critical care management may only need to focus on outcomes that are bespoke to that particular scope; that is, there may be potential for a central COS for critical illness, with additional outcomes relevant to certain interventions or aspects of care.

### Conclusions

Evidence regarding physical rehabilitation for critically ill patients along the recovery continuum continues to grow, albeit limited by the diverse range of outcomes used for evaluating effectiveness. This study has rigorously developed a consensus-generated COS (PRACTICE) for use in future trials to address this particular methodological challenge, containing eight critically important outcomes agreed on by researcher, clinician, and patient and caregiver stakeholder groups. Ascertaining measurement instruments for the PRACTICE core outcomes is now required to facilitate implementation of the COS.

## Supplemental Materials

10.1513/AnnalsATS.202406-581OC Online Data Supplement
