# Supplementary material for: PRACTICE: Development of a Core Outcome Set for Trials of Physical Rehabilitation in Critical Illness
Source: Ann Am Thorac Soc. 2024 Dec 1;21(12):1742–50. doi: 10.1513/AnnalsATS.202406-581OC (PMC11622824; doi:10.1513/AnnalsATS.202406-581OC)
Supplement: Online Data Supplement [file AnnalsATS.202406-581OCS1.docx]

**PRACTICE: Development of a Core Outcome Set for Trials of Physical**

**Rehabilitation in Critical Illness**

Bronwen Connolly, Matthew Barclay, Chantal Davies, Nicholas Hart, Natalie Pattison, Gordon Sturmey, Paula Williamson_,_ Dale M. Needham, Linda Denehy, Bronagh Blackwood

**Online Data Supplement**

**E1. Participant recruitment**

To recruit the participant panel for the modified Delphi consensus study, relevant contact details for each stakeholder group (‘Researchers’, ‘Clinicians’, and Patients/Caregivers’) were identified using national and international critical care organisations, networks, direct contacts, and web-based searches. Organisation leads or individuals (where applicable) were approached directly via an email letter of invitation to participate including detail outlining the study, anticipated timelines for overall commitment, estimated time required for completion of each survey round, and confirming that completion of surveys indicated consent to participate. In all cases, these letters were then circulated within organisations/networks/direct contacts (to facilitate a snowball approach to recruitment) requesting expressions of interest to be made to the lead author (BC). Responses to expressions of interest were supplemented by a detailed Participant Information Sheet.

*‘Researcher’ stakeholder group*

This group comprised members from clinical trial group organisations within InFACT (International Forum of Acute Care Trialists, [www.infactglobal.org](http://www.infactglobal.org)), and senior or corresponding authors from physical rehabilitation publications identified via a systematic review of quantitative literature (1).

*‘Clinician’ stakeholder group*

Clinicians with a primary role in clinical practice and at Consultant (or equivalent) level for medics, or with at least three years specialist critical care experience for nurses and allied health professionals, were recruited from international multi-professional clinical organisations relevant to critical care, and direct networks e.g. physiotherapy, critical care medicine, nursing. Examples of these organisations included: *UK* - Intensive Care Society, Association of Chartered Physiotherapists in Respiratory Care, British Association of Critical Care Nurses, Critical Care Specialty Group (British Dietetics Association); *Europe* - European Society of Intensive Care Medicine, and various profession- and health-services clinician specific subgroups); *North America* – American Physical Therapy Association, American Association of Critical Care Nurses; *Australasia* – Australian Physiotherapy Association, Australia-New Zealand Intensive Care Society, Society of Intensive Care Medicine Singapore; *Global* – International Confederation of Cardiorespiratory Physical Therapists

*‘Patient/caregiver’ stakeholder group*

Former patients and caregivers were recruited from support groups, charities, patient/public engagement groups, and personal contacts.

**E2. Outcomes and descriptors included in Round 1 of the modified Delphi consensus process**

An initial list of outcomes for potential inclusion in the PRACTICE core outcome set (COS) was sourced from systematic reviews of quantitative (1) and qualitative (2) literature, and supported by findings from patient and care partner interviews. Outcomes were de-duplicated from these sources, reviewed, clarified, and informed by the study team, and unique outcomes identified. Descriptors for these outcomes were then developed and iteratively refined following feedback from the study team. Thirty outcomes formed Round 1 of the Delphi consensus (Table E1).

**Table E1.** Outcomes and descriptors included in Round 1 of the modified Delphi consensus process

| **Overarching outcome** | **Definition/explanation** |
| --- | --- |
| Fatigue | An overwhelming, sustained feeling of exhaustion, mental or physical tiredness, having little energy for physical and mental work |
| Activities of daily living | Being able to complete usual everyday tasks including self-care e.g. bathing, dressing or feeding, or other activities e.g. cooking, cleaning, managing finances, or shopping |
| Physical function | How well a person can perform physical functional activities e.g. transferring from sitting to standing or from a bed to chair, walking, climbing stairs, or balance |
| Exercise capacity | Ability to carry out exercise, a person’s level of fitness |
| Emotional and mental wellbeing | A person’s mood, how often they worry, feel anxious, depressed or sad, how often they get angry or upset, and their self esteem |
| Muscle and/or motor nerve function | Feeling weak, sore or numb |
| Joint function | Stiff or aching joints |
| Nutrition-related parameters | Weight change, changes to appetite, changes to taste |
| Pain | Any bodily pain |
| Cognitive function | Problems with someone's memory, concentration, language, thinking and ability to understand instructions |
| Sleep and related symptoms | Trouble falling asleep, trouble staying asleep, changes to sleeping pattern |
| Respiratory (pulmonary) function and symptoms | Trouble breathing, shortness of breath, coughing |
| Gastrointestinal symptoms | Nausea, vomiting, constipation, |
| Urinary function | Incontinence (frequency or urge) |
| Return to work or prior role | Returning to previous job (full or part-time), changing to a different job, doing the same thing you did before being in the ICU |
| Health-related quality of life | How well does a person feel about life; how much is a person’s health affecting their quality of life |
| Delirium and related symptoms | Having an episode of acute confusion, hallucinations, delusions, or nightmares |
| Social roles, activities or relationships | Connecting with others, maintaining friendships and romantic relationships, ability to join in activities with/for others |
| Place of residence | The location where a patient is discharged to following treatment e.g. home, acute care hospital, nursing home, specialist rehabilitation centre, assisted living |
| Survival | Surviving (being alive) after ICU admission, hospital admission or after hospital discharge |
| Duration of mechanical ventilation | Duration of time a patient spends being mechanically ventilated (using a breathing machine) |
| Reintubation | Occasion where replacement of the breathing tube between the patient and the ventilator (breathing machine) is required |
| Successful extubation | Occasion where the breathing tube between the patient and the ventilator (breathing machine) is successfully removed at a specified time point |
| Financial impact on patient | Dealing with hospital bills, lost income from time off work, paying for help with care at home or transportation |
| Healthcare resource utilisation | Seeing more doctors, physio/physical-therapists or other healthcare clinicians, needing to be readmitted to the ICU or hospital, number of days spent in the ICU, hospital or other healthcare setting |
| Patient experience of physical rehabilitation | What did patients think about, and how motivated were they to take part in, physical rehabilitation |
| Frailty | Decreased reserve (capacity) in an individual that may mean a stressful experience could have significant impact on health and wellbeing |
| Swallowing function and symptoms | Trouble swallowing food or liquids |
| Communication difficulties | Problems communicating with others e.g. by speech, writing, or gesture |
| Sexual function | Decreased desire to have sex, inability to have sex |

*Abbreviations:* ICU = intensive care unit

**E3. Revisions to proposed outcomes after Round 1 of the modified Delphi consensus process**

Additional outcomes (n=51) suggested by participants during Round 1 of the modified Delphi consensus process were reviewed by the study team to remove duplicate items and those considered to be beyond the scope of the PRACTICE COS. These additional outcomes, their consideration and rationale, and action for Round 2 are reported in Table E2. During this process, the study team also reviewed comments from participants and consequently felt it necessary to revise the wording of a number of outcomes from Round 1 to enhance clarity. These revisions, plus the descriptors for new outcomes, were subsequently reviewed independently for clarity, sense, and phrasing, and are reported in Table E3 – these outcomes formed Round 2 of the modified Delphi consensus study.

**Table E2.** Additional outcomes, consideration and rationale, and action for Round 2

| **Additional outcome*** | **Consideration; rationale** | **Action for Round 2** |
| --- | --- | --- |
| Bone Health (e.g. bone mineral density; fracture risk) | New outcome, relevant to scope of COS | Include as new outcome ‘Bone health’ |
| Heterotopic ossification | Considered classified under the above | Include as an example in the descriptor for the above outcome |
|  |  |  |
| Resumption of driving | Example of an “instrumental” activity of daily living, and could be considered one component of ‘Activities of daily living’ outcome | Revise existing outcome wording for clarity |
| Living independently and not needing a lot of assistance with day to day tasks | Reflected in outcome ‘Activities of Daily Living’ | Do not include |
|  |  |  |
| Time to first out of bed | Potential measure for ‘Physical Function’ therefore retain for future reference for determining *‘how’*, if ‘Physical Function’ is rated as a core outcome | Do not include |
|  |  |  |
| Sensory changes e.g. vision or hearing | Beyond scope of current core outcome set | Do not include |
|  |  |  |
| Anxiety | Incorporated in existing outcome ‘Emotional and Mental Wellbeing’ | Revise existing wording for clarity |
| Depression | Incorporated in existing outcome ‘Emotional and Mental Wellbeing’ | Revise existing wording for clarity |
| Psychological distress | Incorporated in existing outcome ‘Emotional and Mental Wellbeing’ | Revise existing wording for clarity |
| Evidence of new anxiety/panic attacks | Incorporated in existing outcome ‘Emotional and Mental Wellbeing’ | Revise existing wording for clarity |
| Posttraumatic Stress Disorder | Incorporated in existing outcome ‘Emotional and Mental Wellbeing’ | Revise existing wording for clarity |
|  |  |  |
| Resilience | New outcome, relevant to scope of COS | Include as new outcome ‘Resilience’ |
| Coping | Considered related to the above | Include in descriptor of above outcome |
|  |  |  |
| Medication management | Beyond scope of current core outcome set | Do not include |
| Medication changes | Beyond scope of current core outcome set | Do not include |
|  |  |  |
| Rehospitalisation (unscheduled) | Incorporated in existing outcome ‘Healthcare resource utilisation’ | Revise existing wording for clarity |
| Time in ITU/time on ward pre-discharge from hospital | Incorporated in existing outcome ‘Healthcare resource utilisation’ | Revise existing wording for clarity |
| Time in post hospital rehab setting (skilled nursing facility; LTACH etc) prior to return to original environment | Incorporated in existing outcome ‘Healthcare resource utilisation’ | Revise existing wording for clarity |
| Number of ICU admissions | Incorporated in existing outcome ‘Healthcare resource utilisation’ | Revise existing wording for clarity |
| Readmission to ICU | Incorporated in existing outcome ‘Healthcare resource utilisation’ | Revise existing wording for clarity |
| Length of hospital stay | Incorporated in existing outcome ‘Healthcare resource utilisation’ | Revise existing wording for clarity |
| New requirement of Oxygen prescription or tracheostomy in situ on discharge | Oxygen  Beyond scope of current core outcome set  Tracheostomy  Incorporated in existing outcome ‘Healthcare resource utilisation’ | Do not include  Revise existing wording for clarity |
| Length of time with tracheostomy | Incorporated in existing outcome ‘Healthcare resource utilisation’ | Revise existing wording for clarity |
| Tracheostomy insertion/length in/ combined with ventilation not required etc. | Incorporated in existing outcome ‘Healthcare resource utilisation’ | Revise existing wording for clarity |
| Dependence on ongoing medical treatment | Incorporated in existing outcome ‘Healthcare resource utilisation’ | Revise existing wording for clarity |
|  |  |  |
| Perceptions of family/caregiver | Beyond scope of current core outcome set | Do not include |
| Assist family in accepting patient post ARDS | Beyond scope of current core outcome set | Do not include |
| Family support | Beyond scope of current core outcome set | Do not include |
| Patient’s perceived impact on the carer/loved one? I.e. How the patient dependence has changed pre and post admission? | Beyond scope of current core outcome set | Do not include |
|  |  |  |
| Utility measure | Potential measure for ‘Healthcare resource utilisation’ therefore retain for future reference for determining *‘how’*, if ‘Healthcare resource utilisation’ is rated as a core outcome | Do not include |
|  |  |  |
| Muscle mass | Potential measure for ‘Muscle and/or motor nerve function’ therefore retain for future reference for determining *‘how’*, if ‘Muscle and/or motor nerve function’ is rated as a core outcome | Revise existing outcome name for clarity |
| Muscle strength | Potential measure for ‘Muscle and/or motor nerve function’ therefore retain for future reference for determining *‘how’*, if ‘Muscle and/or motor nerve function’ is rated as a core outcome | Revise existing outcome name for clarity |
| Muscle architecture or muscle quality | Potential measure for ‘Muscle and/or motor nerve function’ therefore retain for future reference for determining *‘how’*, if ‘Muscle and/or motor nerve function’ is rated as a core outcome | Revise existing outcome name for clarity |
| Hand grip | Potential measure for ‘Muscle and/or motor nerve function’ therefore retain for future reference for determining *‘how’*, if ‘Muscle and/or motor nerve function’ is rated as a core outcome | Revise existing outcome name for clarity |
|  |  |  |
| Cognition must be evaluated separately (ex. orientation; attention; etc) | Should ‘Cognitive function’ be rated a core outcome, then further details of specific components will be considered | Revise existing wording for clarity |
| Deficits in organisational skills/attention | Should ‘Cognitive function’ be rated a core outcome, then further details of specific components will be considered | Revise existing wording for clarity |
|  |  |  |
| Body weight change | Incorporated in ‘Nutrition-related parameters’ | Do not include |
|  |  |  |
| Time to reintubation | Potential measure for ‘Reintubation’ therefore retain for future reference for determining *‘how’*, if ‘Reintubation’ is rated as a core outcome | Do not include |
|  |  |  |
| Sitting | Incorporated in existing ‘Physical Function’ outcome | Do not include |
| Balance function | Incorporated in existing ‘Physical Function’ outcome | Do not include |
| Vestibular function | Incorporated in existing ‘Physical Function’ outcome | Do not include |
|  |  |  |
| Body image issues e.g. stoma formation; loss of limb | Beyond scope of current core outcome set | Do not include |
|  |  |  |
| Tissue viability issues | Beyond scope of current core outcome set – relates to general clinical management of patients | Do not include |
| Details about the Physical therapy hospital-based (or ICU-based) protocol (intensity; frequency; duration...) | Beyond scope of current core outcome set – data related to intervention fidelity | Do not include |
| Time dedicated by the patient for physical rehabilitation | Unclear; possibly linked to above regarding specifying details of physical rehabilitation) | Do not include |
| Transfusion of blood and blood products during ICU stay | Beyond scope of current core outcome set – relates to medical management of patients | Do not include |
| Use of steroids during ICU stay | Beyond scope of current core outcome set – relates to medical management of patients | Do not include |
| Diagnosis / patient groups etc. | Not applicable as an outcome – these data would contribute to characterising the population included in a study cohort | Do not include |
| Describe what is the most important change you have noticed since your illness | Limited specificity | Do not include |
| Would the patient repeat the episode of care if needed? | Beyond scope of current core outcome set – relate to considerations about future medical management of patients | Do not include |
| Something like "spiritual development/trust" (we are too much focussed on measurable science!) | Beyond scope of current core outcome set | Do not include |

*Suggestions are reported verbatim from participants. Duplicate items removed and only suggestions with unique wording reported.

**Table E3.** Round 2 outcomes, revised descriptors, and additional new outcomes

| **Overarching outcome** | **Definition/explanation*** |
| --- | --- |
| Fatigue | An overwhelming, sustained feeling of exhaustion, mental or physical tiredness, having little energy for physical and mental work |
| Activities of daily living | Being able to complete usual everyday tasks including personal or self-care e.g. bathing, dressing or feeding, or other activities that help a person live independently in the community e.g. cooking, cleaning, managing finances, shopping, or driving |
| Physical function | How well a person can perform physical functional activities e.g. transferring from sitting to standing or from a bed to chair, walking, climbing stairs, or balance |
| Exercise capacity | Ability to carry out exercise, a person’s level of fitness |
| Emotional and mental wellbeing | How often a person experiences anxiety, depression, post-traumatic stress, sadness, distress, anger, upset, panic attacks, or low self-esteem |
| Muscle and/or motor nerve structure and function | Feeling weak, loss of muscle bulk, muscle ache or soreness |
| Joint function | Stiff or aching joints |
| Nutrition-related parameters | Weight change, changes to appetite, changes to taste |
| Pain | Any bodily pain |
| Cognitive function | Problems with memory, concentration, language, thinking, ability to understand instructions, attention, orientation, organisational skills |
| Sleep and related symptoms | Trouble falling asleep, trouble staying asleep, changes to sleeping pattern |
| Respiratory (pulmonary) function and symptoms | How well the lungs work, trouble breathing, shortness of breath, coughing |
| Gastrointestinal symptoms | Nausea, vomiting, constipation, |
| Urinary function | Incontinence (frequency or urge) |
| Return to work or prior role | Returning to previous job (full or part-time), changing to a different job, doing the same thing you did before being in the ICU |
| Health-related quality of life | How much a person’s health affects their quality of life (how well or satisfied a person feels about life, their well-being); how does a person’s health affect their physical, mental, emotional, and social functioning |
| Delirium and related symptoms | Having an episode of acute confusion, hallucinations, delusions, or nightmares |
| Social roles, activities or relationships | Connecting with others, maintaining friendships and romantic relationships, ability to join in activities with/for others |
| Place of residence | The location where a patient is discharged to following treatment e.g. home, acute care hospital, nursing home, specialist rehabilitation centre, assisted living |
| Survival | Surviving (being alive) after ICU admission, hospital admission or after hospital discharge |
| Duration of mechanical ventilation | Duration of time a patient spends being mechanically ventilated (using a breathing machine) |
| Reintubation | Occasion where replacement of the breathing tube between the patient and the ventilator (breathing machine) is required |
| Successful extubation | Occasion where the breathing tube between the patient and the ventilator (breathing machine) is successfully removed at a specified time point |
| Financial impact on patient | Dealing with hospital bills, lost income from time off work, paying for help with care at home or transportation |
| Healthcare resource utilisation | Seeing more doctors, physio/physical-therapists or other healthcare clinicians. Needing to be readmitted to the ICU or hospital. Number of days spent in the ICU, hospital ward or other healthcare setting e.g. rehabilitation centre |
| Patient experience of physical rehabilitation | What did patients think about, and how motivated were they to take part in, physical rehabilitation |
| Frailty | Decreased reserve (capacity) in an individual that may mean a stressful experience could have significant impact on health and wellbeing |
| Swallowing function and symptoms | Trouble swallowing food or liquids |
| Communication difficulties | Problems communicating with others e.g. by speech, writing, or gesture |
| Sexual function | Decreased desire to have sex, inability to have sex |
| Bone health  (new outcome added) | The quality of a person’s bone structure, their risk of fractures (breaks), and other bone-related conditions e.g. osteoporosis (loss of bone density causing bone weakness), or heterotopic ossification (where bone is present in soft tissues such as muscles, where it is not normally found) |
| Resilience  (new outcome added) | An individual’s ability to deal with psychologically stressful circumstances or a crisis; their ability to cope with difficulties |

*Text in red indicated revised wording of outcomes, and two new outcomes, for Round 2 of the modified Delphi consensus process

**E4. Modified Delphi consensus process method**

We conducted a two-round, international, multi-stakeholder, modified Delphi consensus process using methods similar to those in the development of the other core outcome sets in critical care (3-6). Additional details of the methods are presented.

*Round 1*

At the start of Round 1, demographic data including age, sex, country of residence, duration of clinical and/or research experience (if applicable), and years since ICU discharge (if applicable) were collected, and participants were also asked to select their stakeholder group. Participants were reminded on the purpose of the PRACTICE study, the definition of a core outcome set, and to score outcomes *without* the influence of considering availability, practicality, or psychometric robustness of any instruments available to measure that outcome (6). For each outcome (Table E2), participants then rated its importance for inclusion in the PRACTICE core outcome set, using the Grading of Recommendations Assessment, Development and Evaluation (GRADE) scale (7) as described in the main text. Further options for text-based comments and/or additional suggested outcomes were provided. To minimise potential response order bias (primacy and recency effects (8)) the outcomes were presented in one of four unique, randomised orders (6). Pilot testing of the online system was conducted prior to the launch of Round 1 involving members of the steering group and independent reviewers.

*Round 2*

Only those participants completing Round 1 were invited to complete Round 2. All outcomes from Round 1 were carried through to Round 2, with clarification to descriptors where necessary, plus additional outcomes agreed by the study team (Table E4). Histograms for each outcome from Round 1 were displayed according to aggregate response for the whole participant panel, and per stakeholder group. Participants were also shown their previous individual score for each Round 1 outcome, and were asked to re-score the original based on this feedback, plus the two new additional outcomes added to the list. Where any change of score altered the overall category of importance rating, participants were asked to report the rationale for this change.

Participants were requested to complete survey rounds within 7 days of receipt, and non-responders were contacted via email with reminders. Contact details for the research team were provided within each online survey, and with all correspondence.

The results of the importance scoring for the two additional outcomes in Round 2 were discussed by the study team, for consideration of the need for a third round of scoring for these two outcomes alone (thereby ensuring all outcomes had undergone two rounds of consensus scoring overall); on balance, the results were considered insufficient to indicate potential critical importance for inclusion in the core outcome set (Resilience, 40%; Bone Health, 16%) as to warrant a third round of scoring and the associated burden for participants.

**E5. Detailed participant characteristics**

In total, 329 participants completed Round 1 of the modified Delphi consensus process. Eight clinicians were re-categorised into the ‘Researcher’ stakeholder group, and 4 researchers were re-categorised into the ‘Clinician’ stakeholder group, according to the predefined criteria for each stakeholder group. Detailed participant characteristics are reported in Table E4.

**Table E4.** Detailed participant characteristics

| Characteristic | All participants (n=329) | Researchers (n=58) | Clinicians (n=247) | Patients and caregivers (n=24) |
| --- | --- | --- | --- | --- |
| Country |  |  |  |  |
| UK |  |  |  |  |
| UK | 193 (59) | 15 (26) | 167 (67) | 11 (46) |
| North America |  |  |  |  |
| US | 49 (15) | 10 (17) | 27 (11) | 12 (50) |
| Canada | 2 (<1) | 2 (3) | 0 | 0 |
| Europe |  |  |  |  |
| Austria | 1 (<1) | 0 | 1 (<1) | 0 |
| Belgium | 1 (<1) | 1 (2) | 0 | 0 |
| Czech Republic | 1 (<1) | 0 | 1 (<1) | 0 |
| Denmark | 2 (<1) | 0 | 2 (<1) | 0 |
| France | 12 (4) | 3 (5) | 9 (4) | 0 |
| Germany | 5 (2) | 2 (3) | 3 (1) | 0 |
| Greece | 5 (2) | 2 (3) | 3 (1) | 0 |
| Ireland | 2 (<1) | 1 (2) | 1 (<1) | 0 |
| Italy | 3 (<1) | 3 (5) | 0 | 0 |
| Netherlands | 1 (<1) | 0 | 1 (<1) | 0 |
| Spain | 4 (1) | 2 (3) | 2 (<1) | 0 |
| Sweden | 1 (<1) | 0 (0) | 1 (<1) | 0 |
| Switzerland | 4 (1) | 1 (2) | 3 (1) | 0 |
| Australasia |  |  |  |  |
| Australia | 23 (7) | 9 (16) | 13 (5) | 1 (4) |
| New Zealand | 1 (<1) | 1 (2) | 0 | 0 |
| South America |  |  |  |  |
| Argentina | 2 (<1) | 1 (2) | 1 (<1) | 0 |
| Brazil | 5 (2) | 3 (5) | 2 (<1) | 0 |
| Chile | 1 (<1) | 0 | 1 (<1) | 0 |
| Africa |  |  |  |  |
| South Africa | 6 (2) | 0 | 6 (2) | 0 |
| Asia |  |  |  |  |
| India | 1 (<1) | 0 | 1 (<1) | 0 |
| Pakistan | 1 (<1) | 1 (2) | 0 | 0 |
| Singapore | 2 (<1) | 0 | 2 (<1) | 0 |
| Taiwan | 1 (<1) | 1 (2) | 0 | 0 |
| Professional involvement with patients |  |  |  |  |
| In the ICU | 153 (50) | 32 (55) | 121 (49) | N/A |
| Following transfer to the hospital ward/floor | 3 (1) | 0 | 3 (1) | N/A |
| Following discharge from hospital | 1 (<1) | 0 | 1 (<1) | N/A |
| In the ICU and following transfer to the hospital ward/floor | 77 (25) | 3 (5) | 74 (30) | N/A |
| In the ICU and following discharge from hospital | 19 (6) | 6 (10) | 13 (5) | N/A |
| Following transfer to the hospital ward/floor and following discharge from hospital | 1 (<1) | 1 (2) | 0 | N/A |
| All of the above | 49 (16) | 14 (24) | 35 (14) | N/A |

Data presented as n (%). Note % are rounded to nearest whole, and may not total 100.

*Abbreviations:* ICU = intensive care unit

**E6. Detailed Round 1 scoring breakdown**

Thirty outcomes were included in Round 1 of the modified Delphi consensus process. A detailed breakdown of outcome scoring is presented in Table E5. Outside of the 4 outcome reaching consensus for inclusion in the COS, there was variability in importance rating across the stakeholder groups for remaining outcomes.

Participants indicating ‘Unable to score’ for any outcome was overall very low: Health-related quality of life (n=1 (4%), Patient/caregiver); Physical function (n=1 (<1%), Clinician); Survival (n=1 (4%), Patient/caregiver); Delirium (n=4; n=1 (<1%), Clinician; n=1 (2%), Researcher; n=2 (8%), Patient/caregiver); Duration MV (n=2 (8%), Patient/caregiver); Fatigue (n=1 (2%), Researcher); Financial impact (n=5; n=4 (2%), Clinician; n=1 (4), Patient/caregiver); Frailty (n=4 (2%), Clinician); Gastrointestinal symptoms (n=1 (<1%), Clinician); Healthcare resource utilisation (n=2; n=1 (<1%), Clinician; n=1 (4%), Patient/caregiver); Joint function (n=3; n=1 (<1%), Clinician; n=1 (2%), Researcher; n=1 (4%), Patient/caregiver); Muscle and/or motor nerve function (n=1 (2%), Researcher); Nutrition-related parameters (n=2 (1%), Clinician); Patient experience of rehabilitation (n=1 (4%), Patient/caregiver); Reintubation (n=5; n=1 (2%), Researcher; n=4 (17%), Patient/caregiver); Respiratory symptoms (n=2; n=1 (2%) Researcher; n=1 (4%), Patient/caregiver); Return to work or prior role (n=1 (4%), Patient/caregiver); Sexual function (n=7; n=3 (1%), Clinician; n=2 (3%), Researcher; n=2 (8%), Patient/caregiver); Successful extubation (n=4; n=1 (<1%), Clinician; n=1 (2%), Researcher; n=2 (8%), Patient/caregiver); Swallowing function (n=2; n=1 (<1%), Clinician; n=1 (4%), Patient/caregiver); Urinary function (n=2; n=1 (<1%), Clinician; n=1 (4%), Patient/caregiver).

**Table E5.** Distribution of Round 1 survey scoring

|  |  | **Proportion of all participants scoring each outcome on the GRADE 1-9 scale** | | | | | | | | |
| --- | --- | --- | --- | --- | --- | --- | --- | --- | --- | --- |
| **Outcome** | **Stakeholder**  **Group** | **Not important** | | | **Important but not critical** | | | **Critical** | | |
|  |  | **1** | **2** | **3** | **4** | **5** | **6** | **7** | **8** | **9** |
| **CONSENSUS MET** | | | | | | | | | | |
| Activities of daily living | Clinician | 0 (0%) | 0 (0%) | 1 (<1%) | 2 (1%) | 5 (2%) | 18 (7%) | 53 (21%) | 62 (25%) | 106 (43%) |
|  | Researcher | 0 (0%) | 0 (0%) | 1 (2%) | 1 (2%) | 2 (3%) | 4 (7%) | 11 (19%) | 23 (40%) | 16 (28%) |
|  | Pt/Caregiver | 0 (0%) | 0 (0%) | 1 (4%) | 1 (4%) | 1 (4%) | 2 (8%) | 3 (13%) | 3 (13%) | 13 (54%) |
| Health-related quality of life | Clinician | 0 (0%) | 0 (0%) | 3 (1%) | 7 (3%) | 8 (3%) | 26 (11%) | 85 (34%) | 52 (21%) | 65 (26%) |
|  | Researcher | 0 (0%) | 0 (0%) | 0 (0%) | 0 (0%) | 5 (9%) | 8 (14%) | 10 (17%) | 22 (38%) | 13 (22%) |
|  | Pt/Caregiver | 0 (0%) | 0 (0%) | 0 (0%) | 1 (4%) | 2 (8%) | 3 (13%) | 4 (17%) | 6 (25%) | 7 (29%) |
| Physical function | Clinician | 0 (0%) | 0 (0%) | 0 (0%) | 1 (<1%) | 1 (<1%) | 8 (3%) | 37 (15%) | 52 (21%) | 147 (60%) |
|  | Researcher | 0 (0%) | 0 (0%) | 0 (0%) | 0 (0%) | 1 (2%) | 1 (1.7%) | 7 (12%) | 13 (22%) | 36 (62%) |
|  | Pt/Caregiver | 0 (0%) | 0 (0%) | 0 (0%) | 0 (0%) | 0 (0%) | 3 (13%) | 4 (17%) | 5 (21%) | 12 (50%) |
| Survival | Clinician | 2 (1%) | 0 (0%) | 6 (2%) | 7 (3%) | 15 (6%) | 23 (9%) | 58 (24%) | 34 (14%) | 102 (41%) |
|  | Researcher | 0 (0%) | 0 (0%) | 0 (0%) | 0 (0%) | 4 (7%) | 5 (8.6%) | 12 (21%) | 11 (19%) | 26 (45%) |
|  | Pt/Caregiver | 0 (0%) | 0 (0%) | 1 (4%) | 0 (0%) | 0 (0%) | 0 (0%) | 2 (8%) | 3 (13%) | 17 (71%) |
| **CONSENSUS NOT MET** | | | | | | | | | | |
| Cognitive function | Clinician | 0 (0%) | 0 (0%) | 3 (1%) | 7 (3%) | 12 (5%) | 37 (15%) | 84 (34%) | 42 (17%) | 62 (25%) |
|  | Researcher | 0 (0%) | 0 (0%) | 1 (2%) | 3 (5%) | 2 (3%) | 13 (22%) | 16 (28%) | 14 (24%) | 9 (16%) |
|  | Pt/Caregiver | 0 (0%) | 0 (0%) | 0 (0%) | 3 (13%) | 1 (4%) | 1 (4%) | 5 (21%) | 4 (17%) | 10 (42%) |
| Communication difficulties | Clinician | 0 (0%) | 0 (0%) | 8 (3%) | 15 (6%) | 20 (8%) | 63 (26%) | 78 (32%) | 32 (13%) | 31 (13%) |
|  | Researcher | 0 (0%) | 2 (3%) | 2 (3%) | 5 (9%) | 7 (12%) | 19 (33%) | 13 (22%) | 7 (12%) | 3 (5%) |
|  | Pt/Caregiver | 0 (0%) | 0 (0%) | 0 (0%) | 2 (8%) | 1 (4%) | 0 (0%) | 7 (29%) | 4 (17%) | 10 (42%) |
| Delirium and related symptoms | Clinician | 0 (0%) | 1 (<1%) | 3 (1%) | 15 (6%) | 19 (8%) | 49 (20%) | 70 (28%) | 52 (21%) | 37 (15%) |
|  | Researcher | 0 (0%) | 1 (2%) | 0 (0%) | 4 (7%) | 4 (7%) | 12 (21%) | 18 (31%) | 13 (22%) | 5 (7%) |
|  | Pt/Caregiver | 0 (0%) | 1 (4%) | 0 (0%) | 1 (4%) | 3 (13%) | 3 (13%) | 3 (13%) | 6 (25%) | 5 (21%) |
| Duration of mechanical ventilation | Clinician | 0 (0%) | 1 (<1%) | 2 (1%) | 13 (5%) | 27 (11%) | 40 (16%) | 66 (27%) | 49 (20%) | 49 (20%) |
|  | Researcher | 0 (0%) | 0 (0%) | 0 (0%) | 5 (9%) | 3 (5%) | 10 (17%) | 20 (35%) | 10 (17%) | 10 (17%) |
|  | Pt/Caregiver | 0 (0%) | 0 (0%) | 0 (0%) | 1 (4%) | 2 (8%) | 3 (13%) | 7 (29%) | 2 (8%) | 7 (29%) |
| Emotional and mental wellbeing | Clinician | 0 (0%) | 0 (0%) | 3 (1%) | 13 (5%) | 23 (9%) | 48 (19%) | 79 (32%) | 40 (16%) | 41 (17%) |
|  | Researcher | 0 (0%) | 0 (0%) | 0 (0%) | 2 (3%) | 2 (3%) | 17 (29%) | 27 (47%) | 5 (9%) | 5 (9%) |
|  | Pt/Caregiver | 0 (0%) | 0 (0%) | 0 (0%) | 0 (0%) | 1 (4%) | 6 (25%) | 2 (8%) | 2 (8%) | 13 (54%) |
| Exercise capacity | Clinician | 0 (0%) | 1 (<1%) | 3 (1%) | 11 (5%) | 17 (7%) | 43 (17%) | 75 (30%) | 44 (18%) | 52 (21%) |
|  | Researcher | 0 (0%) | 0 (0%) | 0 (0%) | 1 (2%) | 7 (12%) | 12 (21%) | 23 (40%) | 9 (16%) | 6 (10%) |
|  | Pt/Caregiver | 0 (0%) | 0 (0%) | 0 (0%) | 4 (17%) | 0 (0%) | 4 (17%) | 10 (42%) | 1 (4%) | 5 (21%) |
| Fatigue | Clinician | 0 (0%) | 1 (<1%) | 3 (1%) | 9 (4%) | 19 (8%) | 52 (21%) | 94 (38%) | 43 (17%) | 26 (11%) |
|  | Researcher | 0 (0%) | 1 (2%) | 3 (5%) | 4 (7%) | 4 (7%) | 8 (14%) | 24 (41%) | 12 (21%) | 1 (2%) |
|  | Pt/Caregiver | 0 (0%) | 0 (0%) | 0 (0%) | 3 (13%) | 1 (4%) | 5 (21%) | 4 (17%) | 4 (17%) | 7 (29%) |
| Financial impact on patient | Clinician | 2 (1%) | 7 (3%) | 35 (14%) | 36 (15%) | 34 (14%) | 72 (29%) | 32 (13%) | 19 (8%) | 6 (2%) |
|  | Researcher | 1 (1.7%) | 1 (2%) | 4 (7%) | 8 (14%) | 11 (19%) | 15 (26%) | 7 (12%) | 9 (16%) | 2 (3%) |
|  | Pt/Caregiver | 0 (0%) | 1 (4%) | 1 (4%) | 0 (0%) | 3 (13%) | 6 (25%) | 6 (25%) | 1 (4%) | 5 (21%) |
| Frailty | Clinician | 0 (0%) | 0 (0%) | 5 (2%) | 11 (5%) | 15 (6%) | 53 (22%) | 66 (27%) | 52 (21%) | 41 (17%) |
|  | Researcher | 0 (0%) | 0 (0%) | 3 (5%) | 3 (5%) | 1 (2%) | 13 (22%) | 23 (40%) | 8 (14%) | 7 (12%) |
|  | Pt/Caregiver | 0 (0%) | 0 (0%) | 1 (4%) | 0 (0%) | 0 (0%) | 5 (21%) | 9 (38%) | 3 (13%) | 6 (25%) |
| Gastrointestinal symptoms | Clinician | 2 (1%) | 12 (5%) | 42 (17%) | 46 (19%) | 55 (22%) | 53 (22%) | 26 (11%) | 4 (2%) | 6 (2%) |
|  | Researcher | 2 (3%) | 5 (9%) | 13 (22%) | 10 (17%) | 6 (10%) | 14 (24%) | 7 (12%) | 1 (2%) | 0 (0%) |
|  | Pt/Caregiver | 0 (0%) | 1 (4%) | 2 (8%) | 3 (13%) | 2 (8%) | 6 (25%) | 5 (21%) | 2 (8%) | 3 (12%) |
| Healthcare resource utilisation | Clinician | 0 (0%) | 1 (<1%) | 5 (2%) | 10 (4%) | 31 (13%) | 45 (18%) | 76 (31%) | 39 (16%) | 39 (16%) |
|  | Researcher | 0 (0%) | 0 (0%) | 0 (0%) | 4 (7%) | 11 (19%) | 8 (14%) | 13 (22%) | 13 (22%) | 9 (16%) |
|  | Pt/Caregiver | 0 (0%) | 0 (0%) | 0 (0%) | 0 (0%) | 1 (4%) | 5 (21%) | 4 (17%) | 4 (17%) | 9 (38%) |
| Joint function | Clinician | 0 (0%) | 3 (1%) | 18 (7%) | 37 (15%) | 63 (26%) | 62 (25%) | 41 (17%) | 13 (5%) | 9 (4%) |
|  | Researcher | 1 (2%) | 1 (2%) | 5 (9%) | 8 (14%) | 11 (19%) | 15 (26%) | 11 (19%) | 3 (5%) | 2 (3%) |
|  | Pt/Caregiver | 0 (0%) | 0 (0%) | 2 (8%) | 1 (4%) | 2 (8%) | 6 (25%) | 8 (33%) | 2 (8%) | 2 (8%) |
| Muscle and/or motor nerve function | Clinician | 0 (0%) | 0 (0%) | 8 (3%) | 17 (7%) | 31 (13%) | 48 (19%) | 80 (32%) | 28 (11%) | 35 (14%) |
|  | Researcher | 0 (0%) | 0 (0%) | 2 (3%) | 3 (5%) | 3 (5%) | 19 (33%) | 17 (29%) | 8 (14%) | 5 (9%) |
|  | Pt/Caregiver | 0 (0%) | 0 (0%) | 1 (4%) | 0 (0%) | 1 (4%) | 6 (25%) | 8 (33%) | 3 (13%) | 5 (21%) |
| Nutrition-related parameters | Clinician | 0 (0%) | 1 (<1%) | 21 (9%) | 37 (15%) | 53 (22%) | 53 (22%) | 38 (15%) | 25 (10%) | 17 (7%) |
|  | Researcher | 0 (0%) | 1 (1.7%) | 5 (9%) | 7 (12%) | 16 (28%) | 13 (22%) | 10 (17%) | 4 (7%) | 2 (3%) |
|  | Pt/Caregiver | 0 (0%) | 0 (0%) | 1 (4%) | 5 (21%) | 5 (21%) | 5 (21%) | 2 (8%) | 3 (13%) | 3 (13%) |
| Pain | Clinician | 0 (0%) | 1 (<1%) | 8 (3%) | 14 (6%) | 31 (13%) | 67 (27%) | 75 (30%) | 21 (9%) | 30 (12%) |
|  | Researcher | 1 (1.7%) | 0 (0%) | 1 (2%) | 7 (12%) | 5 (9%) | 17 (29%) | 18 (31%) | 4 (7%) | 5 (9%) |
|  | Pt/Caregiver | 0 (0%) | 0 (0%) | 1 (4%) | 0 (0%) | 2 (8%) | 8 (33%) | 5 (21%) | 3 (13%) | 5 (21%) |
| Patient experience of physical rehabilitation | Clinician | 0 (0%) | 1 (<1%) | 8 (3%) | 21 (9%) | 34 (14%) | 55 (22%) | 84 (34%) | 20 (8%) | 24 (10%) |
|  | Researcher | 0 (0%) | 0 (0%) | 6 (10%) | 3 (5%) | 17 (29%) | 14 (24%) | 6 (10%) | 8 (14%) | 4 (7%) |
|  | Pt/Caregiver | 0 (0%) | 0 (0%) | 0 (0%) | 0 (0%) | 1 (4%) | 5 (21%) | 5 (21%) | 3 (13%) | 9 (38%) |
| Place of residence | Clinician | 0 (0%) | 2 (1%) | 9 (5%) | 11 (5%) | 27 (11%) | 48 (19%) | 78 (32%) | 43 (17%) | 29 (12%) |
|  | Researcher | 0 (0%) | 0 (0%) | 1 (2%) | 4 (7%) | 3 (5%) | 14 (24%) | 17 (29%) | 13 (22%) | 6 (10%) |
|  | Pt/Caregiver | 0 (0%) | 0 (0%) | 0 (0%) | 1 (4%) | 2 (8%) | 8 (33%) | 3 (13%) | 2 (8%) | 8 (33%) |
| Reintubation | Clinician | 4 (2%) | 2 (1%) | 14 (6%) | 27 (11%) | 35 (14%) | 53 (22%) | 72 (29%) | 17 (7%) | 23 (9%) |
|  | Researcher | 0 (0%) | 1 (2%) | 5 (9%) | 3 (5%) | 17 (29%) | 11 (19%) | 11 (19%) | 5 (9%) | 4 (7%) |
|  | Pt/Caregiver | 0 (0%) | 1 (4%) | 0 (0%) | 1 (4%) | 3 (13%) | 4 (17%) | 5 (21%) | 2 (8%) | 4 (17%) |
| Respiratory (pulmonary) function and symptoms | Clinician | 0 (0%) | 0 (0%) | 2 (1%) | 11 (5%) | 23 (9%) | 56 (23%) | 76 (31%) | 40 (16%) | 39 (16%) |
|  | Researcher | 0 (0%) | 0 (0%) | 2 (3%) | 7 (12%) | 9 (16%) | 12 (21%) | 15 (26%) | 6 (10%) | 6 (10%) |
|  | Pt/Caregiver | 0 (0%) | 0 (0%) | 0 (0%) | 0 (0%) | 1 (4%) | 2 (8%) | 6 (25%) | 4 (17%) | 10 (42%) |
| Return to work or prior role | Clinician | 1 (<1%) | 0 (0%) | 4 (2%) | 6 (2%) | 21 (9%) | 35 (14%) | 87 (35%) | 56 (23%) | 37 (15%) |
|  | Researcher | 0 (0%) | 0 (0%) | 0 (0%) | 2 (3%) | 0 (0%) | 20 (35%) | 14 (24%) | 13 (22%) | 9 (16%) |
|  | Pt/Caregiver | 1 (4%) | 0 (0%) | 0 (0%) | 1 (4%) | 1 (4%) | 8 (33%) | 8 (33%) | 3 (13%) | 1 (4%) |
| Sexual function | Clinician | 9 (4%) | 6 (2%) | 38 (15%) | 52 (21%) | 48 (19%) | 58 (24%) | 21 (9%) | 6 (2%) | 6 (2%) |
|  | Researcher | 2 (3%) | 6 (10%) | 7 (12%) | 9 (16%) | 11 (19%) | 14 (24%) | 5 (9%) | 1 (2%) | 1 (2%) |
|  | Pt/Caregiver | 0 (0%) | 2 (8%) | 3 (13%) | 1 (4%) | 4 (17%) | 6 (25%) | 5 (21%) | 0 (0%) | 1 (4%) |
| Sleep and related symptoms | Clinician | 1 (<1%) | 1 (<1%) | 5 (2%) | 27 (11%) | 41 (17%) | 77 (31%) | 57 (23%) | 22 (9%) | 16 (7%) |
|  | Researcher | 0 (0%) | 1 (2%) | 2 (3%) | 11 (19%) | 9 (16%) | 16 (28%) | 9 (16%) | 9 (16%) | 1 (2%) |
|  | Pt/Caregiver | 0 (0%) | 0 (0%) | 1 (4%) | 2 (8%) | 2 (8%) | 4 (17%) | 8 (33%) | 3 (13%) | 4 (17%) |
| Social roles; activities or relationships | Clinician | 2 (1%) | 2 (1%) | 17 (7%) | 28 (11%) | 41 (17%) | 52 (21%) | 55 (22%) | 29 (12%) | 21 (9%) |
|  | Researcher | 0 (0%) | 2 (3%) | 3 (5%) | 8 (13.8%) | 9 (16%) | 15 (26%) | 14 (24%) | 5 (9%) | 2 (3%) |
|  | Pt/Caregiver | 0 (0%) | 0 (0%) | 2 (8%) | 0 (0%) | 3 (13%) | 8 (33%) | 6 (25%) | 0 (0%) | 5 (21%) |
| Successful extubation | Clinician | 2 (0.8%) | 3 (1%) | 19 (8%) | 31 (13%) | 32 (13%) | 45 (18%) | 61 (25%) | 23 (9%) | 30 (12%) |
|  | Researcher | 0 (0%) | 0 (0%) | 6 (10%) | 4 (7%) | 6 (10%) | 16 (28%) | 15 (26%) | 5 (9%) | 5 (9%) |
|  | Pt/Caregiver | 0 (0%) | 0 (0%) | 0 (0%) | 0 (0%) | 1 (4%) | 4 (17%) | 4 (17%) | 3 (13%) | 10 (42%) |
| Swallowing function and symptoms | Clinician | 1 (<1%) | 1 (<1%) | 6 (2%) | 20 (8%) | 24 (10%) | 60 (24%) | 79 (32%) | 32 (13%) | 23 (9%) |
|  | Researcher | 0 (0%) | 1 (2%) | 3 (5%) | 6 (10%) | 5 (9%) | 13 (22%) | 17 (29%) | 10 (17%) | 3 (5%) |
|  | Pt/Caregiver | 0 (0%) | 0 (0%) | 0 (0%) | 0 (0%) | 0 (0%) | 5 (21%) | 6 (25%) | 6 (25%) | 6 (25%) |
| Urinary function | Clinician | 3 (1%) | 11 (5%) | 44 (18%) | 43 (17%) | 50 (20%) | 57 (23%) | 27 (11%) | 4 (2%) | 7 (3%) |
|  | Researcher | 4 (7%) | 3 (5%) | 10 (17%) | 5 (9%) | 10 (17%) | 19 (33%) | 5 (9%) | 2 (3%) | 0 (0%) |
|  | Pt/Caregiver | 0 (0%) | 0 (0%) | 0 (0%) | 2 (8%) | 1 (4%) | 8 (33%) | 6 (25%) | 3 (13%) | 3 (13%) |

Data are reported as n (%). % may exceed 100 due to rounding. Participants in each stakeholder group: Clinicians = 247; Researchers = 58; Patients/Caregivers = 24. *Each outcome was scored according to the Grading of Recommendations Assessment, Development and Evaluation (GRADE) scale (7), ranging 1-9 in terms of importance for inclusion in the final core outcome set (1-3, not important for inclusion; 4-6, important but not critical; 7-9, critical for inclusion). Consensus for inclusion of an outcome by a particular stakeholder group was defined as ≥70% of responses rating the outcome as ‘critical’, and less than or equal to 15% of responses rating the outcome ≤3. Consensus for an outcome included in the core outcome set was defined as all three stakeholder groups scoring the outcome as critical for inclusion.

**E7. Detailed Round 2 scoring breakdown**

Thirty-two outcomes were included in Round 2 of the modified Delphi consensus process. A detailed breakdown of outcome scoring is presented in Table E6. Eight outcomes were rated as critically important for inclusion in the COS by all three stakeholder groups; these outcomes form the COS. Seven outcomes scored critical for importance (≥70% overall) by one or two stakeholder groups but did not reach consensus; duration of mechanical ventilation, return to work or prior role, fatigue, respiratory symptoms, healthcare resource utilisation, delirium, and place of residence.

Participants indicating ‘Unable to score’ for any outcome was overall very low: Bone health (n=3; n=1 (<1%), Clinician; n=2 (11%), Patient/caregiver); Delirium (n=3; n=1 (<1%), Clinician; n=1 (2%), Researcher; n=1 (5%) Patient/caregiver); Duration of mechanical ventilation (n=2 (11%), Patient/caregiver); Fatigue (n=1 (2%), Researcher); Financial impact (n=5 (2%), Clinician); Gastrointestinal symptoms (n=1 (<1%), Clinician); Healthcare utilisation (n=1 (5%), Patient/caregiver); Joint function (n=3; n=1 (<1%), Clinician; n=1 (2%), Researcher; n=1 (5%) Patient/caregiver); Muscle and nerve function (n=1 (2%), Researcher); Physical function (n=1 (<1%), Clinician); Reintubation (n=2 (11%), Patient/caregiver); Resilience (n=2; n=1 (<1%), Clinician; n=1 (5%) Patient/caregiver); Respiratory symptoms (n=1 (2%), Researcher); Return to work or prior role (n=1 (5%), Patient/caregiver); Sexual function (n=4; n=2 (1%), Clinician; n=1 (2%), Researcher; n=1 (5%) Patient/caregiver); Successful extubation (n=2 (11%), Patient/caregiver); Survival (n=2; n=1 (<1%), Clinician; n=1 (5%) Patient/caregiver); Resilience (n=1 (<1%), Clinician); Swallowing function (n=1 (<1%), Clinician); Urinary function (n=2; n=1 (<1%), Clinician; n=1 (5%) Patient/caregiver).

**Table E6.** Distribution of Round 2 survey scoring

|  |  | **Proportion of all participants scoring each outcome on the GRADE 1-9 scale** | | | | | | | | |
| --- | --- | --- | --- | --- | --- | --- | --- | --- | --- | --- |
| **Outcome** | **Stakeholder**  **Group** | **Not important** | | | **Important but not critical** | | | **Critical** | | |
|  |  | **1** | **2** | **3** | **4** | **5** | **6** | **7** | **8** | **9** |
| **CONSENSUS MET** | | | | | | | | | | |
| Activities of daily living | Clinician | 0 (0%) | 0 (0%) | 0 (0%) | 0 (0%) | 0 (0%) | 2 (1%) | 35 (15%) | 56 (25%) | 133 (59%) |
|  | Researcher | 0 (0%) | 0 (0%) | 0 (0%) | 0 (0%) | 0 (0%) | 1 (2%) | 6 (11%) | 20 (36%) | 28 (51%) |
|  | Pt/Caregiver | 0 (0%) | 0 (0%) | 1 (5%) | 0 (0%) | 1 (5%) | 0 (0%) | 3 (16%) | 3 (16%) | 11 (58%) |
| Health-related quality of life | Clinician | 0 (0%) | 0 (0%) | 0 (0%) | 2 (1%) | 6 (3%) | 9 (4%) | 90 (40%) | 63 (28%) | 56 (25%) |
|  | Researcher | 0 (0%) | 0 (0%) | 0 (0%) | 0 (0%) | 2 (4%) | 6 (11%) | 11 (20%) | 22 (40%) | 14 (25%) |
|  | Pt/Caregiver | 0 (0%) | 0 (0%) | 0 (0%) | 0 (0%) | 0 (0%) | 4 (21%) | 2 (11%) | 4 (21%) | 9 (47%) |
| Physical function | Clinician | 0 (0%) | 0 (0%) | 0 (0%) | 0 (0%) | 0 (0%) | 0 (0%) | 13 (6%) | 31 (14%) | 181 (80%) |
|  | Researcher | 0 (0%) | 0 (0%) | 0 (0%) | 0 (0%) | 0 (0%) | 0 (0%) | 3 (5%) | 8 (15%) | 44 (80%) |
|  | Pt/Caregiver | 0 (0%) | 0 (0%) | 0 (0%) | 0 (0%) | 0 (0%) | 0 (0%) | 3 (16%) | 3 (16%) | 13 (68%) |
| Survival | Clinician | 2 (1%) | 0 (0%) | 2 (1%) | 4 (2%) | 6 (3%) | 7 (3%) | 43 (19%) | 33 (15%) | 128 (57%) |
|  | Researcher | 0 (0%) | 0 (0%) | 0 (0%) | 0 (0%) | 0 (0%) | 1 (2%) | 12 (22%) | 10 (18%) | 32 (58%) |
|  | Pt/Caregiver | 0 (0%) | 0 (0%) | 0 (0%) | 0 (0%) | 0 (0%) | 0 (0%) | 1 (5%) | 2 (11%) | 15 (79%) |
| Cognitive function | Clinician | 0 (0%) | 0 (0%) | 3 (1%) | 3 (1%) | 1 (<1%) | 25 (11%) | 95 (42%) | 44 (19%) | 55 (24%) |
|  | Researcher | 0 (0%) | 0 (0%) | 0 (0%) | 2 (4%) | 2 (4%) | 10 (18%) | 19 (35%) | 12 (22%) | 10 (18%) |
|  | Pt/Caregiver | 0 (0%) | 0 (0%) | 0 (0%) | 1 (5%) | 1 (5%) | 1 (5%) | 4 (21%) | 2 (11%) | 10 (53%) |
| Exercise capacity | Clinician | 0 (0%) | 1 (<1%) | 1 (<1%) | 2 (1%) | 6 (3%) | 24 (11%) | 119 (53%) | 42 (19%) | 31 (14%) |
|  | Researcher | 0 (0%) | 0 (0%) | 0 (0%) | 1 (2%) | 1 (2%) | 8 (15%) | 33 (60%) | 8 (15%) | 4 (7%) |
|  | Pt/Caregiver | 0 (0%) | 0 (0%) | 0 (0%) | 1 (5%) | 2 (11%) | 5 (26%) | 4 (21%) | 2 (11%) | 5 (26%) |
| Emotional and mental wellbeing | Clinician | 0 (0%) | 0 (0%) | 2 (1%) | 2 (1%) | 13 (6%) | 31 (14%) | 89 (39%) | 46 (20%) | 43 (19%) |
|  | Researcher | 0 (0%) | 0 (0%) | 0 (0%) | 2 (4%) | 1 (2%) | 11 (20%) | 29 (53%) | 7 (13%) | 5 (9%) |
|  | Pt/Caregiver | 0 (0%) | 0 (0%) | 0 (0%) | 0 (0%) | 0 (0%) | 4 (21%) | 3 (16%) | 1 (5%) | 11 (58%) |
| Frailty | Clinician | 0 (0%) | 0 (0%) | 1 (<1%) | 5 (2%) | 9 (4%) | 43 (19%) | 98 (43%) | 48 (21%) | 23 (10%) |
|  | Researcher | 0 (0%) | 0 (0%) | 0 (0%) | 3 (5%) | 2 (4%) | 9 (16%) | 31 (56%) | 7 (13%) | 3 (5%) |
|  | Pt/Caregiver | 0 (0%) | 0 (0%) | 0 (0%) | 0 (0%) | 0 (0%) | 1 (5%) | 11 (58%) | 2 (11%) | 5 (26%) |
| **CONSENSUS NOT MET** | | | | | | | | | | |
| Communication difficulties | Clinician | 0 (0%) | 0 (0%) | 3 (1%) | 9 (4%) | 11 (5%) | 63 (28%) | 84 (37%) | 30 (13%) | 26 (12%) |
|  | Researcher | 0 (0%) | 0 (0%) | 2 (4%) | 3 (5%) | 6 (11%) | 17 (31%) | 17 (31%) | 8 (15%) | 2 (4%) |
|  | Pt/Caregiver | 0 (0%) | 0 (0%) | 0 (0%) | 0 (0%) | 1 (5%) | 1 (5%) | 5 (26%) | 4 (21%) | 8 (42%) |
| Delirium and related symptoms | Clinician | 0 (0%) | 0 (0%) | 2 (1%) | 7 (3%) | 9 (4%) | 45 (20%) | 88 (39%) | 49 (22%) | 25 (11%) |
|  | Researcher | 0 (0%) | 0 (0%) | 0 (0%) | 3 (5%) | 3 (5%) | 10 (18%) | 23 (42%) | 11 (20%) | 4 (7%) |
|  | Pt/Caregiver | 0 (0%) | 1 (5%) | 0 (0%) | 0 (0%) | 0 (0%) | 6 (32%) | 2 (11%) | 4 (21%) | 5 (26%) |
| Duration of mechanical ventilation | Clinician | 0 (0%) | 1 (<1%) | 1 (<1%) | 10 (4%) | 13 (6%) | 25 (11%) | 93 (41%) | 47 (21%) | 36 (16%) |
|  | Researcher | 0 (0%) | 0 (0%) | 0 (0%) | 1 (2%) | 3 (5%) | 9 (16%) | 26 (47%) | 10 (18%) | 6 (11%) |
|  | Pt/Caregiver | 0 (0%) | 0 (0%) | 0 (0%) | 0 (0%) | 0 (0%) | 5 (26%) | 6 (32%) | 1 (5%) | 5 (26%) |
| Fatigue | Clinician | 0 (0%) | 0 (0%) | 0 (0%) | 4 (2%) | 9 (4%) | 41 (18%) | 117 (52%) | 36 (16%) | 19 (8%) |
|  | Researcher | 0 (0%) | 1 (2%) | 0 (0%) | 1 (2%) | 0 (0%) | 6 (11%) | 35 (64%) | 11 (20%) | 0 (0%) |
|  | Pt/Caregiver | 0 (0%) | 0 (0%) | 0 (0%) | 1 (5%) | 2 (11%) | 5 (26%) | 4 (21%) | 2 (11%) | 5 (26%) |
| Financial impact on patient | Clinician | 1 (<1%) | 4 (2%) | 19 (8%) | 33 (15%) | 38 (17%) | 94 (42%) | 21 (9%) | 7 (3%) | 4 (2%) |
|  | Researcher | 1 (2%) | 1 (2%) | 1 (2%) | 5 (9%) | 9 (16%) | 23 (42%) | 10 (18%) | 4 (7%) | 1 (2%) |
|  | Pt/Caregiver | 0 (0%) | 0 (0%) | 0 (0%) | 0 (0%) | 2 (11%) | 5 (26%) | 7 (37%) | 2 (11%) | 3 (16%) |
| Gastrointestinal symptoms | Clinician | 0 (0%) | 7 (3%) | 43 (19%) | 37 (16%) | 67 (30%) | 50 (22%) | 16 (7%) | 3 (1%) | 2 (1%) |
|  | Researcher | 1 (2%) | 5 (9%) | 13 (24%) | 5 (9%) | 12 (22%) | 14 (25%) | 4 (7%) | 1 (2%) | 0 (0%) |
|  | Pt/Caregiver | 1 (5%) | 0 (0%) | 1 (5%) | 3 (16%) | 4 (21%) | 4 (21%) | 4 (21%) | 1 (5%) | 1 (5%) |
| Healthcare resource utilisation | Clinician | 0 (0%) | 1 (<1%) | 1 (<1%) | 4 (2%) | 20 (9%) | 38 (17%) | 94 (42%) | 41 (18%) | 27 (12%) |
|  | Researcher | 0 (0%) | 0 (0%) | 0 (0%) | 0 (0%) | 8 (15%) | 8 (15%) | 17 (31%) | 15 (27%) | 7 (13%) |
|  | Pt/Caregiver | 0 (0%) | 0 (0%) | 0 (0%) | 0 (0%) | 2 (11%) | 3 (16%) | 4 (21%) | 1 (5%) | 8 (42%) |
| Joint function | Clinician | 0 (0%) | 1 (<1%) | 8 (4%) | 27 (12%) | 65 (29%) | 82 (36%) | 33 (15%) | 5 (2%) | 4 (2%) |
|  | Researcher | 0 (0%) | 0 (0%) | 5 (9%) | 6 (11%) | 11 (20%) | 20 (36%) | 11 (20%) | 1 (2%) | 0 (0%) |
|  | Pt/Caregiver | 0 (0%) | 0 (0%) | 1 (5%) | 1 (5%) | 0 (0%) | 7 (37%) | 7 (37%) | 1 (5%) | 1 (5%) |
| Muscle and/or motor nerve function | Clinician | 0 (0%) | 0 (0%) | 0 (0%) | 7 (3%) | 20 (9%) | 48 (21%) | 107 (47%) | 25 (11%) | 19 (8%) |
|  | Researcher | 0 (0%) | 0 (0%) | 0 (0%) | 1 (2%) | 4 (7%) | 12 (22%) | 26 (47%) | 8 (15%) | 3 (5%) |
|  | Pt/Caregiver | 0 (0%) | 0 (0%) | 1 (5%) | 0 (0%) | 1 (5%) | 3 (16%) | 9 (47%) | 1 (5%) | 4 (21%) |
| Nutrition-related parameters | Clinician | 0 (0%) | 1 (<1%) | 9 (4%) | 37 (16%) | 72 (32%) | 63 (28%) | 24 (11%) | 13 (6%) | 7 (3%) |
|  | Researcher | 0 (0%) | 1 (2%) | 2 (4%) | 7 (13%) | 19 (35%) | 17 (31%) | 8 (15%) | 1 (2%) | 0 (0%) |
|  | Pt/Caregiver | 0 (0%) | 0 (0%) | 1 (5%) | 5 (26%) | 3 (16%) | 3 (16%) | 3 (16%) | 3 (16%) | 1 (5%) |
| Pain | Clinician | 0 (0%) | 1 (<1%) | 3 (1%) | 8 (4%) | 22 (10%) | 72 (32%) | 88 (39%) | 17 (8%) | 15 (7%) |
|  | Researcher | 1 (2%) | 0 (0%) | 0 (0%) | 3 (5%) | 5 (9%) | 22 (40%) | 16 (29%) | 5 (9%) | 3 (5%) |
|  | Pt/Caregiver | 0 (0%) | 0 (0%) | 0 (0%) | 0 (0%) | 2 (11%) | 6 (32%) | 7 (37%) | 1 (5%) | 3 (16%) |
| Patient experience of physical rehabilitation | Clinician | 0 (0%) | 1 (<1%) | 4 (2%) | 11 (5%) | 20 (9%) | 63 (28%) | 93 (41%) | 15 (7%) | 19 (8%) |
|  | Researcher | 0 (0%) | 0 (0%) | 1 (2%) | 4 (7%) | 12 (22%) | 16 (29%) | 13 (24%) | 7 (13%) | 2 (4%) |
|  | Pt/Caregiver | 0 (0%) | 0 (0%) | 0 (0%) | 0 (0%) | 2 (11%) | 3 (16%) | 5 (26%) | 1 (5%) | 8 (42%) |
| Place of residence | Clinician | 0 (0%) | 0 (1%) | 2 (1%) | 6 (3%) | 11 (5%) | 47 (21%) | 105 (46%) | 34 (15%) | 21 (9%) |
|  | Researcher | 0 (0%) | 0 (0%) | 0 (0%) | 1 (2%) | 3 (5%) | 13 (24%) | 26 (47%) | 7 (13%) | 5 (9%) |
|  | Pt/Caregiver | 0 (0%) | 0 (0%) | 0 (0%) | 1 (5%) | 1 (5%) | 5 (26%) | 4 (21%) | 3 (16%) | 5 (26%) |
| Reintubation | Clinician | 1 (<1%) | 1 (<1%) | 10 (4%) | 17 (8%) | 34 (15%) | 51 (23%) | 92 (41%) | 10 (4%) | 10 (4%) |
|  | Researcher | 0 (0%) | 1 (2%) | 1 (2%) | 3 (5%) | 14 (25%) | 11 (20%) | 20 (36%) | 3 (5%) | 2 (4%) |
|  | Pt/Caregiver | 0 (0%) | 1 (5%) | 0 (0%) | 0 (0%) | 0 (0%) | 6 (32%) | 7 (37%) | 3 (16%) | 0 (0%) |
| Respiratory (pulmonary) function and symptoms | Clinician | 0 (0%) | 0 (0%) | 0 (0%) | 4 (2%) | 12 (5%) | 38 (17%) | 111 (49%) | 37 (16%) | 24 (11%) |
|  | Researcher | 0 (0%) | 0 (0%) | 0 (0%) | 4 (7%) | 8 (15%) | 11 (20%) | 19 (35%) | 10 (18%) | 2 (4%) |
|  | Pt/Caregiver | 0 (0%) | 0 (0%) | 0 (0%) | 0 (0%) | 0 (0%) | 1 (5%) | 5 (26%) | 3 (16%) | 10 (53%) |
| Return to work or prior role | Clinician | 0 (0%) | 0 (0%) | 0 (0%) | 1 (<1%) | 13 (6%) | 31 (14%) | 123 (54%) | 37 (16%) | 24 (11%) |
|  | Researcher | 0 (0%) | 0 (0%) | 0 (0%) | 0 (0%) | 2 (4%) | 15 (27%) | 24 (44%) | 10 (18%) | 2 (4%) |
|  | Pt/Caregiver | 1 (2%) | 0 (0%) | 0 (0%) | 0 (0%) | 1 (5%) | 6 (32%) | 6 (32%) | 3 (16%) | 1 (5%) |
| Sexual function | Clinician | 5 (2%) | 1 (<1%) | 29 (13%) | 47 (21%) | 56 (25%) | 67 (30%) | 13 (6%) | 3 (1%) | 3 (1%) |
|  | Researcher | 0 (0%) | 5 (9%) | 7 (13%) | 9 (16%) | 11 (20%) | 16 (29%) | 4 (7%) | 1 (2%) | 1 (2%) |
|  | Pt/Caregiver | 1 (5%) | 2 (11%) | 2 (11%) | 1 (5%) | 2 (11%) | 5 (26%) | 4 (21%) | 0 (0%) | 1 (5%) |
| Sleep and related symptoms | Clinician | 0 (0%) | 0 (0%) | 4 (2%) | 15 (7%) | 30 (13%) | 92 (41%) | 61 (27%) | 16 (7%) | 8 (4%) |
|  | Researcher | 1 (2%) | 0 (0%) | 1 (2%) | 6 (11%) | 11 (20%) | 19 (35%) | 10 (18%) | 7 (13%) | 0 (0%) |
|  | Pt/Caregiver | 0 (0%) | 0 (0%) | 2 (11%) | 2 (11%) | 1 (5%) | 5 (26%) | 5 (26%) | 2 (11%) | 2 (11%) |
| Social roles; activities or relationships | Clinician | 1 (<1%) | 0 (0%) | 10 (4%) | 12 (5%) | 37 (16%) | 76 (34%) | 59 (26%) | 20 (9%) | 11 (5%) |
|  | Researcher | 0 (0%) | 1 (2%) | 2 (4%) | 8 (15%) | 6 (11%) | 23 (42%) | 13 (24%) | 1 (2%) | 1 (2%) |
|  | Pt/Caregiver | 0 (0%) | 0 (0%) | 1 (5%) | 1 (5%) | 1 (5%) | 5 (26%) | 7 (37%) | 0 (0%) | 4 (21%) |
| Successful extubation | Clinician | 1 (<1%) | 2 (1%) | 7 (3%) | 21 (9%) | 29 (13%) | 55 (24%) | 74 (33%) | 17 (8%) | 20 (9%) |
|  | Researcher | 0 (0%) | 0 (0%) | 1 (2%) | 4 (7%) | 5 (9%) | 20 (36%) | 17 (31%) | 6 (11%) | 2 (4%) |
|  | Pt/Caregiver | 0 (0%) | 0 (0%) | 0 (0%) | 0 (0%) | 1 (5%) | 5 (26%) | 2 (11%) | 5 (26%) | 4 (21%) |
| Swallowing function and symptoms | Clinician | 1 (<1%) | 0 (0%) | 1 (<1%) | 8 (4%) | 19 (8%) | 54 (24%) | 101 (45%) | 25 (11%) | 16 (7%) |
|  | Researcher | 1 (2%) | 0 (0%) | 1 (2%) | 4 (7%) | 6 (11%) | 9 (16%) | 24 (44%) | 7 (13%) | 3 (5%) |
|  | Pt/Caregiver | 0 (0%) | 0 (0%) | 0 (0%) | 0 (0%) | 0 (0%) | 3 (16%) | 6 (32%) | 5 (26%) | 5 (26%) |
| Urinary function | Clinician | 1 (<1%) | 6 (3%) | 39 (17%) | 35 (15%) | 50 (22%) | 73 (32%) | 15 (7%) | 4 (2%) | 2 (1%) |
|  | Researcher | 2 (4%) | 2 (4%) | 8 (15%) | 7 (13%) | 9 (16%) | 24 (44%) | 2 (4%) | 1 (2%) | 0 (0%) |
|  | Pt/Caregiver | 0 (0%) | 0 (0%) | 0 (0%) | 2 (11%) | 0 (0%) | 8 (42%) | 5 (26%) | 2 (11%) | 1 (5%) |
| **ADDITIONAL OUTCOMES IN ROUND 2** | | | | | | | | | | |
| Resilience | Clinician | 1 (<1%) | 0 (0%) | 9 (4%) | 9 (4%) | 40 (18%) | 78 (35%) | 53 (23%) | 30 (13%) | 5 (2%) |
|  | Researcher | 1 (2%) | 0 (0%) | 4 (7%) | 8 (15%) | 10 (18%) | 13 (24%) | 16 (29%) | 2 (4%) | 1 (2%) |
|  | Pt/Caregiver | 0 (0%) | 0 (0%) | 0 (0%) | 0 (0%) | 2 (11%) | 2 (11%) | 4 (21%) | 4 (21%) | 6 (32%) |
| Bone Health | Clinician | 1 (<1%) | 1 (<1%) | 11 (5%) | 37 (16%) | 66 (29%) | 77 (34%) | 30 (13%) | 1 (<1%) | 1 (<1%) |
|  | Researcher | 2 (4%) | 0 (0%) | 5 (9%) | 10 (18%) | 16 (29%) | 12 (22%) | 7 (13%) | 3 (5%) | 0 (0%) |
|  | Pt/Caregiver | 0 (0%) | 0 (0%) | 0 (0%) | 2 (11%) | 2 (11%) | 6 (32%) | 5 (26%) | 0 (0%) | 2 (11%) |

Data are reported as n (%). % may exceed 100 due to rounding. Participants in each stakeholder group: Clinicians = 226; Researchers = 55; Patients/Caregivers = 19. *Each outcome was scored according to the Grading of Recommendations Assessment, Development and Evaluation (GRADE) scale (7), ranging 1-9 in terms of importance for inclusion in the final core outcome set (1-3, not important for inclusion; 4-6, important but not critical; 7-9, critical for inclusion). Consensus for inclusion of an outcome by a particular stakeholder group was defined as ≥70% of responses rating the outcome as ‘critical’, and less than or equal to 15% of responses rating the outcome ≤3. Consensus for an outcome included in the core outcome set was defined as all three stakeholder groups scoring the outcome as critical for inclusion.

Construct validity is not mandated or suggested as part of core outcome set development (9). However, to determine if the consensus core outcomes reflected those found in the literature, we planned a nested methodological study in the original protocol (10), to answer the question: Which outcomes in the final PRACTICE core outcome set originated from which information sources?

We mapped the final core outcomes to the systematic reviews of quantitative and qualitative literature, and the qualitative interviews with patients and caregivers, which had collectively informed the initial list of outcomes for the Delphi consensus process. Table E7 summarises this process to determine which sources the core outcomes featured in. Six of the core outcomes were evident in each of the three sources (physical function, activities of daily living, health-related quality of life, exercise capacity, cognitive function, and emotional and mental wellbeing), one outcome was evidence in two of the sources (survival), and one outcome was evident in one of the sources (frailty).

**Table E7.** Summary of sources of core outcomes in the PRACTICE core outcome set

| **Outcome** | **SR of quantitative literature** | **SR of qualitative literature** | **Qualitative interviews** |
| --- | --- | --- | --- |
| Physical Function | X | X | X |
| Activities of Daily Living | X | X | X |
| Survival | X |  | X |
| Health-related Quality of Life | X | X | X |
| Exercise Capacity | X | X | X |
| Cognitive Function | X | X | X |
| Emotional and Mental Wellbeing | X | X | X |
| Frailty | X |  |  |

**REFERENCES**

1. Connolly B, Allum L, Granger CL, Mortimore J, Denehy L, Williamson P, Hart N, Blackwood B. Measuring the Metrics of Outcomes in Trials of Physical Rehabilitation During Critical Illness and Recovery; A Systematic Review. *Am J Respir Crit Care Med* 2017; 195: A7107.

2. Connolly B, Mortimore J, Apps C, Crowley C, Corner E, Pattison N. The patient experience of recovery from critical illness: a systematic review and meta-synthesis of qualitative studies across the continuum of recovery. *Intensive Care Medicine Experimental* 2018; 6: 0836.

3. Blackwood B, Ringrow S, Clarke M, Marshall JC, Connolly B, Rose L, McAuley DF. A Core Outcome Set for Critical Care Ventilation Trials. *Crit Care Med* 2019; 47: 1324-1331.

4. Needham D, Sepulveda K, Dinglas V, Chessare C, Friedman L, Bingham III C, Turnbull A. Core Outcome Measures for Clinical Research in Acute Respiratory Failure Survivors. An International Modified Delphi Consensus Study. *Am J Respir Crit Care Med* 2017; 196: 1122-1130.

5. Rose L, Agar M, Burry LD, Campbell N, Clarke M, Lee J, Siddiqi N, Page VJ. Development of core outcome sets for effectiveness trials of interventions to prevent and/or treat delirium (Del-COrS): study protocol. *BMJ Open* 2017; 7.

6. Turnbull AE, Sepulveda KA, Dinglas VD, Chessare CM, Bingham COI, Needham DM. Core Domains for Clinical Research in Acute Respiratory Failure Survivors: An International Modified Delphi Consensus Study. *Crit Care Med* 2017; 45: 1001-1010.

7. Guyatt G, Oxman A, Kunz R, Atkins D, Brozek J, Vist G, Alderson P, Glasziou P, Falck-Ytter Y, Schunemann H. GRADE guidelines: 2. Framing the question and deciding on important outcomes. *J Clin Epidemiol* 2011; 64: 395-400.

8. Lavrakas P. Response Order Effects. *In: Encylopedia of Survey Research Methods* 2008; Thousand Oaks, CA, Sage Publications. Available at <http://methods.sagepub.com/Reference//encyclopedia-of-survey-research-methods/n397.xml>. Accessed 13th August, 2018.

9. Williamson PR, Altman DG, Bagley H, Barnes KL, Blazeby JM, Brookes ST, Clarke M, Gargon E, Gorst S, Harman N, Kirkham JJ, McNair A, Prinsen CAC, Schmitt J, Terwee CB, Young B. The COMET Handbook: version 1.0. *Trials* 2017; 18: 280.

10. Connolly B, Denehy L, Hart N, Pattison N, Williamson P, Blackwood B. Physical Rehabilitation Core Outcomes In Critical illness (PRACTICE): protocol for development of a core outcome set. *Trials* 2018; 19: 294.
